# Consequences of irradiation on blood-brain tumor barrier model of Diffuse Midline Glioma: characterization of physical and metabolic properties

**DOI:** 10.1186/s12987-026-00778-6

**Published:** 2026-02-24

**Authors:** Marine Carroué, Eloise Happernegg, Flavie Perrot, Marie-Christine Boucau, Lucie Dehouck, Emmanuel Sevin, Mélanie Arcicasa, Joanne Balsamelli, Fumitaka Shimizu, Takashi Kanda, Angel M. Carcaboso, Robert-Alain Toillon, Maxime Culot, Samuel Meignan, Fabien Gosselet, Caroline Mysiorek

**Affiliations:** 1 Univ. Artois, UR 2465, Laboratoire de la Barrière Hémato-Encéphalique (LBHE), Lens, F-62300 France; 2 Univ. Lille, CNRS, Inserm, CHU Lille, Institut de Recherche Contre Le Cancer de Lille, UMR9020 - UMR-S 1277 - Canther Cancer Heterogeneity, Plasticity and Resistance to Therapies, Lille, F-59000 France; 3 Tumorigenesis and Resistance to Treatment Unit, Centre Oscar Lambret, Lille, 59000 France; 4 Department of Neurology and Clinical Neuroscience, Yamaguchi University Graduate School of Medicine, Ube, Japan; 5 Department of Neurology, Neuromuscular Center Yoshimizu Hospital, Shimonoseki, Japan; 6 Pediatric Cancer Program, Institut de Recerca Sant Joan de Deu (IRSJD), Hospital Sant Joan de Deu, Esplugues de Llobregat, Barcelona, 08950 Spain

**Keywords:** Blood-tumor barrier model, Blood-brain barrier, Endothelial cells, Pericytes, Radiotherapy, DMG, DIPG, Brain tumor, Inflammation, Resistance to treatment, Efflux pumps, MFSD2A

## Abstract

**Background:**

Diffuse midline glioma (DMG) is a rare and aggressive pediatric brain tumor, with a median survival of less than 12 months. Due to its location in the pons, surgical resection is impossible, leaving radiation therapy as the only palliative treatment option. Unfortunately, radiation therapy yields minimal improvement in survival. Thus, characterization of the vascular component of the DMG microenvironment at the cellular and molecular levels following radiotherapy to improve therapeutic strategies.

**Methods:**

A human syngeneic blood-brain tumor barrier (BBTB) in vitro model, comprising endothelial cells, pericytes and DMG cells was submitted to a single dose of radiation (2 Gγ to 6 Gγ) and was characterized for its physical and metabolic properties over a period of 7 days post-exposure. The results were then compared to the effects of the same irradiation protocol on a physiologic blood-brain barrier (BBB) model.

**Results:**

Following irradiation, the endothelial permeability of the BBB ECs and the BBTB ECs was preserved for up to 7 days but associated with Claudin-5 heterogeneous distribution at the ECs borders and decrease of expression after irradiation. Nevertheless, irradiation was found to potentiate the effect of TNFα on the physical integrity of the BBB, which was less important for the BBTB. The metabolic properties of the BBB and BBTB were modulated by radiation at the transcriptional level. Interestingly, different regulations were observed in endothelial cells and pericytes. Notably, pericytes have demonstrated compensatory effects. Immunoblots confirmed the decrease of BCRP, MRP4 and MFSD2A in BBTB endothelial cells after irradiation. Despite significant reduced efficiency, P-gp/BCRP efflux pump activity remains functional in endothelial cells and pericytes following irradiation.

**Conclusions:**

Irradiation sensitizes the BBB, but to a lesser extent the BBTB, to the effects of pro-inflammatory cytokines. The observed decrease in P-gp/BCRP activity, as well as the involvement of MFSD2A, MRP4 and Claudin-5 regulation, warrant further investigations.

**Supplementary Information:**

The online version contains supplementary material available at 10.1186/s12987-026-00778-6.

## Background

Brain tumors are the most frequently diagnosed solid neoplasms in children and are the leading cause of disease-associated mortality in the pediatric population [1, 2]. High-grade gliomas account for 9.3% of pediatric gliomas and are responsible of 43.8% of cancer related deaths [3]. Among these, diffuse midline glioma (DMG), is a grade IV tumor, representing the second most common brain tumor [4, 5]. Frequently diagnosed in the pons, the DMG (previously named diffuse intrinsic pontine glioma - DIPG) exhibits a diffuse growth pattern, which renders this tumor ineligible for surgical resection. Hence, the DMG remains associated with a median survival of less than 12 months [6]. Despite over 250 clinical trials have been conducted in the last decade, using radiotherapy alone or associated with cytotoxic chemotherapy, none of them have proven to be effective since they have failed to demonstrate any significant improvement in the clinical outcomes [7, 8]. Hence, radiotherapy remains the standard of care for DMG patients, but unfortunately, only for palliative purposes [8–10]. Recently, evaluating cancer treatments in the preclinical steps, by taking into account not only cancer cells but also including the tumor microenvironment (TME), has gained recognition and emerged as an evidence-based strategy for the evaluation of treatment response [11]. In the brain, the blood-brain barrier (BBB) controls exchanges between the blood and the brain parenchyma to maintain brain homeostasis and represents the vascular component of the TME. The BBB restricts the access of neurotoxic compounds but also of xenobiotics and chemotherapeutics. In addition, the BBB allows the delivery of nutrients essential for nervous cells function. Located at the level of endothelial cells (ECs) of brain capillaries, the BBB forms a dynamic barrier, whose properties throughout life are finely regulated by communications at the level of the neurovascular unit (NVU) thanks to chemical mediators or extracellular vesicles [12]. The NVU is made up of ECs, brain pericytes (hBPs) (embedded in the ECs basement membrane), astrocytes, neurons and glial cells [13, 14]. To ensure its barrier properties, the BBB ECs possess physical properties (presence of tight junctions between ECs) and metabolic properties (efflux pumps and drug metabolizing enzymes) to limit the passage of compounds [15, 16]. However, the BBB is permeable and selective, thanks to specific receptors and transporters, to support neuronal function [17, 18]. Renamed blood-brain tumor barrier (BBTB) in the vicinity of the tumor, the BBTB limits the access of therapeutics to tumor cells and is involved in the drug resistance mechanism [19, 20]. Hence, characterising at the cellular and molecular levels the TME is prime of importance. The present study aimed to characterised the physical and metabolic properties of the BBB and the BBTB following radiation exposure. To do so, human syngeneic in vitro models consisting of ECs, hBPs and astrocytes or DMG cells for the BBB model and BBTB model respectively were used [21]. A follow-up of the physical and metabolic properties was done until 7 days post-irradiation in ECs and hBPs. The results presented in this study aimed at improving the understanding of the consequences of radiation on the BBTB at the cellular and molecular levels.

## Materials and methods

### Culture of human cells

The human endothelial cells (ECs) were derived from CD34 + hematopoietic stem cells isolated from human umbilical cord blood and differentiated according to the method described by Pedroso et al. [22]. Written and informed consent was obtained from the donor’s parents for the collection of umbilical cord blood, in compliance with the French Legislation. This protocol was approved by the French Ministry of Higher Education and Research (reference: CODECOH DC2011-1321) and by the local investigational review board (Béthune Maternity Hospital, Beuvry, France). The ECs were cultured in ECs medium (PromoCell medium, ECGMV2, supplemented with Mix C-39226, Heidelberg, Germany) supplemented with 0.5% gentamicin sulfate (10 mg/ml; Biowest, Nuaillé, France).

The Human Brain Pericytes (hBPs), which were kindly provided by Professor Takashi Kanda (Department of Neurology and Clinical Neuroscience, Yamaguchi University Graduate School of Medicine, Ube, Japan), were isolated from human cerebral capillaries, and immortalized through the transfection of the SV40 virus T antigen under heat-sensitive control and human telomerase. The hBPs are cultured in DMEM (Dulbecco’s Modified Eagle Medium; 31600-083, Gibco™) medium supplemented with 4.5 g/L of glucose, 10% inactivated FCS (Fetal Calf Serum F7524, Sigma Aldrich), 1% L-glutamine (Merck) and 1% penicillin-streptomycin antibiotic (15140, Gibco™) [23].

Human Astrocytes isolated from human brainstem (HA-bs, 1840, Sciencell) were cultured in Astrocyte Medium (1801, Sciencell), supplemented with 2% FCS, 1% Astrocyte Growth Supplement (AGS, 1852, Sciencell) and 1% penicillin-streptomycin. To facilitate cell attachment, the culture flasks were coated with Poly-L-Lysine (PLL, 0413, ScienCell) at a concentration of 2µg/cm^2^.

The DMG cell lines were patient-derived cells provided by Dr. Angel Montero Carcaboso (Institut de Recerca Sant Joan de Deu, Barcelona, Spain). The HSJD-DIPG-007 and HSJD-DIPG-013 cell lines (Table 1) were obtained under an Institutional Review Board-approved protocol and with written informed consent (M-1608-C) at Hospital Sant Joan de Deu Barcelona (HSJD), Spain. The cells were cultured as spheres in DMEM/F12 and Neurobasal A medium (1: 1) (11320-074 and 10888-022, Gibco™), containing 1% 1 M HEPES (15630-049, Gibco™), 1% MEM non-essential amino acids (11140-035, Gibco™), 1% 100 mM sodium pyruvate (11360-039, Gibco™), 1% GlutaMAX (35050-038, Gibco™) and 1% antibiotic-antimycotic (15240096, Gibco™), supplemented extemporaneously with 2% B27 without vitamin A (12587010,Gibco™), 20 ng/ml human EGF (AF-100-15), 20 ng/ml human FGF (AF-100-18B), 10 ng/ml human PDGF-AA (100–13 A), 10 ng/ml human PDGF-BB (100-14B, (all growth factors from PeproTech) and 2 µg/ml heparin (H3149-10KU, Sigma-Aldrich).

**Table 1:** Clinical and molecular characteristics of DMG cells

Cells	Type of sample	Sex	Age at diagnosis (years)	Tumor location	H3 mutation	ACVR1 mutation	TP53 mutation
HSJD-DIPG-007	Necropsy	M	10	Pons	*H3F3A* K27M	R206H	wild type
HSJD-DIPG-013	Biopsy	F	6	Pons	H3F3A K27M	wild type	R248Q

### BBB and BBTB models

The syngeneic human BBB and BBTB models were developed according to the protocols described by *Rizzi et al.*,2021 [24] and *Deligne et al.*,*2020* [21], which were derived from the initial procotol of Ceccheli et al. [25]. Shortly, hBPs were seeded at a density of 4.46 × 10^4^ cells/cm^2^ on the reverse side of the Transwell™ insert (TW 12-well, 0.4 μm; Corning Inc., New York, NY, USA) precoated with rat tail type I collagen (10 µg/cm^2^ ; 354236, Corning, New York, USA). After 3 h at 37 °C, the inserts were flipped over to seed the ECs at 7.14 × 10^4^ cells/cm^2^ on the other side of the filter coated with Matrigel™ (354230, Corning, New York, USA). The BBB phenotype is induced in ECs thanks to the co-culture of ECs with hBPs. The ECs are then renamed hBLECs (human Brain Like Endothelial Cells) [25]. To set up the triple culture of the BBB and the BBTB models (BBTB-007 and BBTB-013), the TW inserts were transferred to wells containing astrocytes or DMG cells (HSJD-DIPG-007 or HSJD-DIPG-013) respectively. The two DMG cell lines are widely used in DMG research field and are well characterised [26]. To do so, astrocytes or DMG cells were seeded 24 h before triple culture, at 3.6 × 10^5^ cells/well. The triple culture was performed using ECs medium (5% CO_2_, 37 °C) which was renewed every two days. The triple culture lasted 7 days. In order to simplify the nomenclature, hBLECs will be named BBB ECs or BBTB ECs thought all the manuscript.

### Treatments

#### Radiation exposure

After 7 days of triple culture, the BBB and BBTB models were exposed to X-ray radiation using a surface irradiation device (Xstrahl 100). The single doses (from 2 to 6 Gy (Gray)) were delivered at a rate of 0.8 Gγ/min. The non-irradiated controls were subjected to the same conditions but without irradiation. Following exposure, the triple culture medium was changed every two days. The follow-up of radiation was performed at 2 h, 24 h and 7 days post-exposure.

#### Inflammatory treatment

To mimic an inflammatory condition, 5ng/ml of the pro-inflammatory cytokine, TNFα was added to the cerebral compartment of BBB and BBTB-007 models in serum free ECGMV2 medium containing 0.1% BSA (Sigma-Aldrich) for 24 h [27–29]. 

### Endothelial cells permeability to BBB integrity markers

The physical integrity of the ECs was evaluated by measuring the paracellular diffusion of Lucifer Yellow (LY; 457.25 g/mol; Sigma Aldrich) or radiolabeled ^14^C-saccharose (Perkin ELMER, Boston, USA).

To do so, the filters were transferred to new 12-well plates containing 1.5 ml of Ringer-HEPES solution (RH; 150 mM NaCl, 5.2 mM KCl, 2.2 mM CaCl_2_, 0.2 mM MgCl_2_ 6H_2_O, 6 mM NaHCO_3_, 5 mM HEPES, 2.8 mM glucose; pH 7.4). Then, in each filter, the culture medium was replaced by 0.5 ml of RH-Lucifer Yellow solution at 50µM or radiolabeled ^14^C-Saccharose. The assay was performed at 37 °C. Diffusion of the compound was measured using a 20, 40 and 60 min kinetic. After the kinetic period, the fluorescence of the initial solution, the solution in the filters and in the wells were quantified using the Synergy™ H1 reader (BioTek Instruments, Winooski, USA, excitation/emission wavelengths: 432/538 nm). For the C^14^ isotope, the decay per minute is measured every 90 s using LabLogic 300 SL HIDEX scintillation counter and processing software MikroWin 300 SL.

The permeability coefficient (P*e*) was determined using the following clearance principle calculation: Clearance = A/B where A represents the amount of compound detected in the lower compartment and B the concentration in the upper compartment. Cleared volumes were plotted versus time. The slope of the straight line gives the value of P*St* (“total permeability x surface area”) and P*Sf* (“filter permeability x surface area”). P*St* corresponds to filter permeability + ECs + Matrigel™ + type I collagen + hBPs x surface area, and PSf to filter permeability + Matrigel™ + type I collagen + human hBPs x surface area. P*Se* (“endothelial permeability x surface area”) is then calculated using the formula 1/PSe = 1/PSt − 1/PSf.

The P*e* was then determined by the following calculation: PSe value divided by the filter surface (1.12 cm²) and expressed in cm/min.

### Immunofluorescent staining

Immunofluorescent stainings were performed on fixed cells. According to the targeted protein, the cells were fixed with a 4% or 1% paraformaldehyde fixative solution (PFA) followed by permeabilization with 0.1% Triton X-100 (for γ-H2AX, ZO-1) or cold methanol (for VE-cadherin, Claudin-5).

After several rinses with Phosphate-Buffered Saline Calcium and Magnesium Free (PBS-CMF; 136 mM NaCl, 2.7 mM KCl, 1.5 mM KH_2_PO_4_, 8mM Na_2_PO_4_ 12H_2_O), pericytes were gently scraped off the filter and non-specific sites were blocked with pure Fish Serum Blocking buffer (SBB, 37527, Thermo Scientific, Rockford, USA). For ECs staining, the filters were incubated with primary antibody solution for 1 h (diluted in PBS-CMF containing 5% SBB) for ZO-1 (rabbit anti-ZO-1, 61-7300, Invitrogen), VE-Cadherin (rabbit anti-VE-cadherin, ab33168, Abcam), Claudin-5 (rabbit anti-CLDN-5, 34-1600, Invitrogen), phospho-Histone H2A.X (mouse anti-phospho-Histone H2A.X (Ser139) monoclonal antibody (3F2), MA1-2022, Invitrogen).

After three rinses with PBS-CMF 5% SBB, the cells were incubated with secondary antibody in the dark for 30 min (Goat anti-rabbit Alexa fluor 568, A11036; Alexa fluor 680, A32734 Invitrogen, Oregon, USA; or Goat anti-mouse Alexa fluor 680, A32729, Invitrogen).

The staining of the nuclei was performed using DAPI reagent (diluted 1:1000 in PBS-CMF) (D1306, Invitrogen). Finally, after several washes with PBS-CMF, the filters were mounted under a coverslip using Prolong™ Diamond Antifade Mountant (P36961, Invitrogen, Oregon, USA) and observed thanks to a Leica DMi8 fluorescence microscope and LASX Premium software (Leica Microsystems, Wetzlar, Germany).

### RT-qPCR

The cell lysates were collected after two washing steps with RH at 4 °C. Firstly, hBPs were collected from the reverse side of the filter, followed by ECs.

Cell lysates were collected with RP1 lysis buffer supplemented with 10mM of dithiothreitol (DTT, 161–0611, BioRad) and mRNA were extracted using a NucleoSpin^®^ RNA/protein kit (Macherey-Nagel, Dueren, Germany), according to the manufacturer’s protocol. Concentration and purity of isolated mRNA were assessed by spectrophotometric assay using the Synergy™ H1 and Take 3 plate (BioTek Instruments). Sample purity was determined by the absorbance ratio, 260 nm (nucleic acids)/280 nm (proteins), which must be between 1.9 and 2.1. Samples were then stored at -80 °C.

The Reverse transcription (RT) step was performed using a thermocycler (MJ Research, Reno, USA) to obtain cDNA from 250ng of total RNA. The reaction mix contains a reverse transcriptase enzyme from the iScript™ Reverse Transcription Supermix kit (1708841, BioRad, Hercules, USA). The enzyme was used according to the manufacturer’s protocol. To detect possible contamination of samples with genomic DNA, enzyme-free negative controls were performed. The reverse transcription step (20 min at 46 °C) was preceded by a primer hybridization step (5 min at 25 °C) and followed by an enzyme inactivation step (1 min at 95 °C). Samples were stored at -20 °C.

The Supermix SsoFast™ EvaGreen^®^ kit (172–5201, BioRad) containing DNA polymerase, cofactors, dNTPs and DNA intercalant EvaGreen, was used for the quantitative PCR (qPCR) to amplify the cDNA obtained. Amplification can be monitored in real time using EvaGreen technology, with the CFX96 thermal cycler (BioRad): programmed for enzyme activation and denaturation at 95 °C and 40 cycles for primers hybridization at 60 °C. Cq values were obtained for each gene tested, using the Bio-Rad CFX Manager software, and target gene expression levels were calculated using the 2^−ΔΔCt^ method and normalized with the GAPDH reference gene (Table 2).

**Table 2:** Nucleotide sequences for human primers forward (F) and reverse (R) used for the qPCR analysis (synthesized by Invitrogen)

Target	Gene	Primer F/*R*	Primers Sequences	Accession Number
GAPDH	*GAPDH*	F	GAT GAC ATC AAG AAG GTG GTG A	NM_001357943.2
		R	GCT GTT GAA GTC AGA GGA GAC C	
P-gp	*ABCB1*	F	CAG ACA GCA GCT GAC AGT CCA AGA ACA GGA CT	NM_001348946.2
		R	GCC TGG CAG CTG GAA GAC AAA TAC ACA AAA TT	
BCRP	*ABCG2*	F	TGG CTG TCA TGG CTT GAG TA	NM_001348986.2
		R	GCC ACG TGA TTC TTC CAC AA	
MRP1	*ABCC1*	F	GTC CTT AAA CAA GGA GGA GGA CAC G	NM_001438720.1
		R	TCC TTG GAG GAG TAC ACA ACC T	
MRP2	*ABCC2*	F	GGA GAA GTT CTG CAA CTC TAC TT	NM_000392.5
		R	TAG GTA GCC CAA GGG AAT CCA	
MRP4	*ABCC4*	F	CCC GTG TAC CAG GAG GTG AA	NM_001301830.2
		R	GGG ATT GAG CCA CCA GAA GAA	
MRP5	*ABCC5*	F	CCC AGT CCT GGG TAT AGA AGT G	NM_001023587.3
		R	CGA GTT CTC CTG AAC TTG GAA T	
CYP1A1	*CYP1A1*	F	TTT TAC ATC CCC AAG GGG CG	NM_001319217.2
		R	TCT CAC CGA TAC ACT TCC GC	
CYP1B1	*CYP1B1*	F	CCA CTG AAG TGG CCT AAC CC	NM_000104.4
		R	TTT TCG CAG GCT CAT TTG GG	
CYP2S1	*CYP2S1*	F	GAT CTA CAG CCC CTG TTC GG	NM_030622.8
		R	TTA ATT CCC AAG CCG GAC CC	
CYP2D6	*CYP2D6*	F	TCA TCA CCA ACC TGT CAT CGG	NM_001025161.3
		R	CCT CCG GCT TCA CAA AGT GG	
MFSD2A	*MFSD2A*	F	CAG CAT CCT CCA AAG CAC TGA A	NM_032793.5
		R	CAA GTG CAT AGC AAA GCT TGT TG	

### Western blot analysis

Prior to protein extraction, the cells were rinsed twice with cold RH solution. All steps were performed at 4 °C. Firstly, hBPs were mechanically collected from the bottom side of the filter. Then the cell lysates were collected using RIPA 1X lysis buffer (20–188, Merck Millipore, Burlington, USA) supplemented with 1% of protease and phosphatase inhibitors (P8340, P5726 and P0044; Sigma Aldrich). Samples are kept on ice for 1 h and vortexed before undergoing a freeze/thaw step at -20 °C. After a centrifugation of the cell lysate (10 min, 10 000 rpm, 4 °C), the protein contents of the supernatant were quantified using Bradford method (5000006, Biorad, Hercules, USA).

Western blot assays were performed using 20 µg of total protein mixed with Laemmli loading buffer (1610747, Bio-Rad) supplemented with β-mercaptoethanol (M-3148, Sigma Aldrich). Following electrophoresis, a wet transfer of proteins was performed on nitrocellulose blotting membranes (0.45 μm NC 10600003, Cytiva Amersham™ Protran). Membranes undergo a 90 min saturation step in a solution of Tris-buffered saline with 0.1% Tween 20 (TBST: 20 mM Tris-HCl pH 7.5, 500 mM NaCl, 0.1% Tween 20; Bio-Rad) containing 5% milk and 3% of goat serum. Then the membranes were incubated with the primary antibody (Table 3) diluted in 1% milk TBS-Tween solution for 2 h at room temperature (RT) (20 min for anti- β-actin primary antibody), or overnight at 4 °C.

After incubation, the membranes were rinsed several times with TBST before incubation for 1 h at RT with the horseradish peroxidase (HRP)-coupled secondary antibody (Table 4). After successive rinses, the chemiluninescence was revealed using an ECL kit (RPN2236, Amersham-cytiva) and detected with the Azure c600 imaging system (Azure Biosystems, Dublin, Ireland). ImageJ software was used for analysis. Protein quantification was calculated relative to β-actin or GAPDH protein.

**Table 3:** Primary antibodies used for Western blot analysis

Protein Target	Name of target	Antibody reference	Host species	Sample condition	Dilution
β–ACTIN	Actin Beta	A5441 (Sigma Aldrich)	Mouse		1 : 20 000
GAPDH	Glyceraldehyde-3-phosphate dehydrogenase	GTX627408 (GeneTex)	Mouse		1 : 1000
BCRP	Breast-Cancer-Resistance Protein	ab207732 (Abcam)	Rabbit	Without heat denaturation + reduction with β − mercaptoethanol	1 : 1000
P-gp	P-glycoprotein	C219 clone GTX23364 (GeneTex)	Mouse		1 : 400
MRP1	Multidrug-resistance-associated Proteins 1	ab260038 (Abcam)	Rabbit		1 : 1000
MRP4	Multidrug-Resistance-associated Proteins 4	ab15602 (Abcam)	Rat		1 : 2000
MFSD2A	Major Facilitator Superfamily Domain-Containing Protein A	SAB3500576 (Sigma Aldrich)	Rabbit	Denaturation at 95 °C for 5 min + reduction with β − mercaptoethanol	1 : 1000
CLDN-5	Claudin-5	GTX49370 (GeneTex)	Rabbit		1 : 1000
ZO-1	Zonula Occludens	61-7300 (Invitrogen)	Rabbit		1 : 500

**Table 4:** Secondary antibody used for Western Blot analysis

Protein Target	Antibody reference	Dilution
anti-mouse- HRP	P0447 (Dako)	1 : 5000 (1 : 10 000 for actin)
anti-rabbit-HRP	P0448 (Dako)	1 : 8000
Anti-rat-HRP	ADI-SAB-220-0500 (Enzo)	1 : 5000

### Efflux pump activity

P-gp/ BCRP activity was assessed in ECs on a filter by measuring intracellular accumulation of Rhodamine 123 (R123). After gentle removal of the hBPs from the bottom side of the filter, the filters containing ECs were placed in 12-well plates filled with RH 0.1% HSA solution (Sigma-Aldrich), and incubated for 2 h at 37 °C with 0.5 ml RH-HSA solution containing 5µM R123 (Sigma Aldrich) with or without 0.5 µM of elacridar, a P-gp/ BCRP inhibitor (Sigma Aldrich). After incubation, the filters were rinsed once, transferred to a new plate, and incubated for an additional 30 min at 37 °C with 0.5 ml RH-HSA^14^C-saccharose with or without elacridar. After incubation, the ECs supernatants were collected for permeability measurement. Cells were rinsed several times with cold RH solution before the collection of the cell lysate using RIPA 1X buffer (Merck Millipore, Burlington, USA). The amount of R123 accumulated in the cells was measured directly in the cell lysates using the Synergy™ H1 instrument (excitation/emission wavelengths: 501/538 nm). The results obtained were normalized by the proteins. The results were expressed as the concentration of R123 (nM) accumulated per µg of protein.

Efflux pump activity of P-gp/ BCRP in hBPs was assessed by real-time Rhodamine 123 pump out assay according to the method described by Sevin et al.,2019 [30]. To do so, hBPs were incubated for 2 h at 37 °C with R123 solution at 20µM. Then the cells were washed twice with RH solution at room temperature and incubated with or without Verapamil solution at 50µM a P-gp/ BCRP inhibitor. The efflux of R123 in the medium was directly monitored every 2 min during 90 min at 37 °C by fluorescent measurement at 501/538nm (BioTek Cytation C10 Confocal Imaging Reader, Agilent). The results in RFU/min/ng proteins were expressed as a percentage relative to the control.

### Cell density measurement and DNA double-strand breaks positive cell count

The counting of ECs nuclei was done on multiple fields of each filter by using the count option on the BioTek Cytation C10 Confocal Imaging Reader and Gene5 software. For the optimal count, the parameters of minimal sizes and thresholds were optimized and fixed for all analysed images.

The anti-γ-H2AX antibody was used to study the induction of DNA double-strand breaks (DSB) in cell nucleus after irradiation. The number of positive cells was determined manually using ImageJ software.

### Mitochondrial activity assays

The MTT tetrazolium reduction assay was used to assess the mitochondrial activity of endothelial cells. Seven days after irradiation, the cell culture medium was changed with 0.5 ml of MTT solution (3-(4,5-Dimethyl-2-thiazolyl)-2,5-diphenyl-2 H-tetrazolium Bromide) (Sigma-Aldrich) at a concentration of 1 mg/ml, the cells were incubated at 37 °C until formation of purple crystals. Crystals formation results from the reduction of tetrazolium to formazan by mitochondrial succinate dehydrogenase of metabolically active cells. Pure DMSO (dimethyl sulfoxide) was used to solubilize intracellular formazan crystals to measure the absorbance at 570 and 630 nm to determined cell activity rate and background subtraction. The results were expressed as a percentage to non-irradiated control.

### Statistical analyses

Individual wells or inserts of cultured cells that underwent the same experimental procedure are defined as replicates. For immunocytochemistry, individual fields of view (4–5 fields of view per culture insert) are defined as replicates. All key experiments were repeated independently and detailed information about error bars, sample size, replication strategy and statistical tests are provided in each figure legend.

The statistical analyses were performed with GraphPad Prism 9.0 software (GraphPad Software Inc, San Diego, CA, USA). When certain outliers are observed using a graphical representation of the results, they are examined using Grubbs test (alpha = 0.05), to confirm their outlier status. The variables were analysed using the Shapiro-Wilk test on the residus to confirm normality. The results were expressed as the mean ± SEM and analysed with Student’s t-test or one-way ANOVA test followed by multiple comparisons tests.

Statistical analysis of dose-response could not be performed using linear regression curves due to a limitation related to sample size.

The threshold for statistical significance was set to *p-value < 0.05, **p < 0.01, ***p < 0.001, ****p < 0.0001.

## Results

### Induction of DNA double-strand breaks

**Fig. 1:**
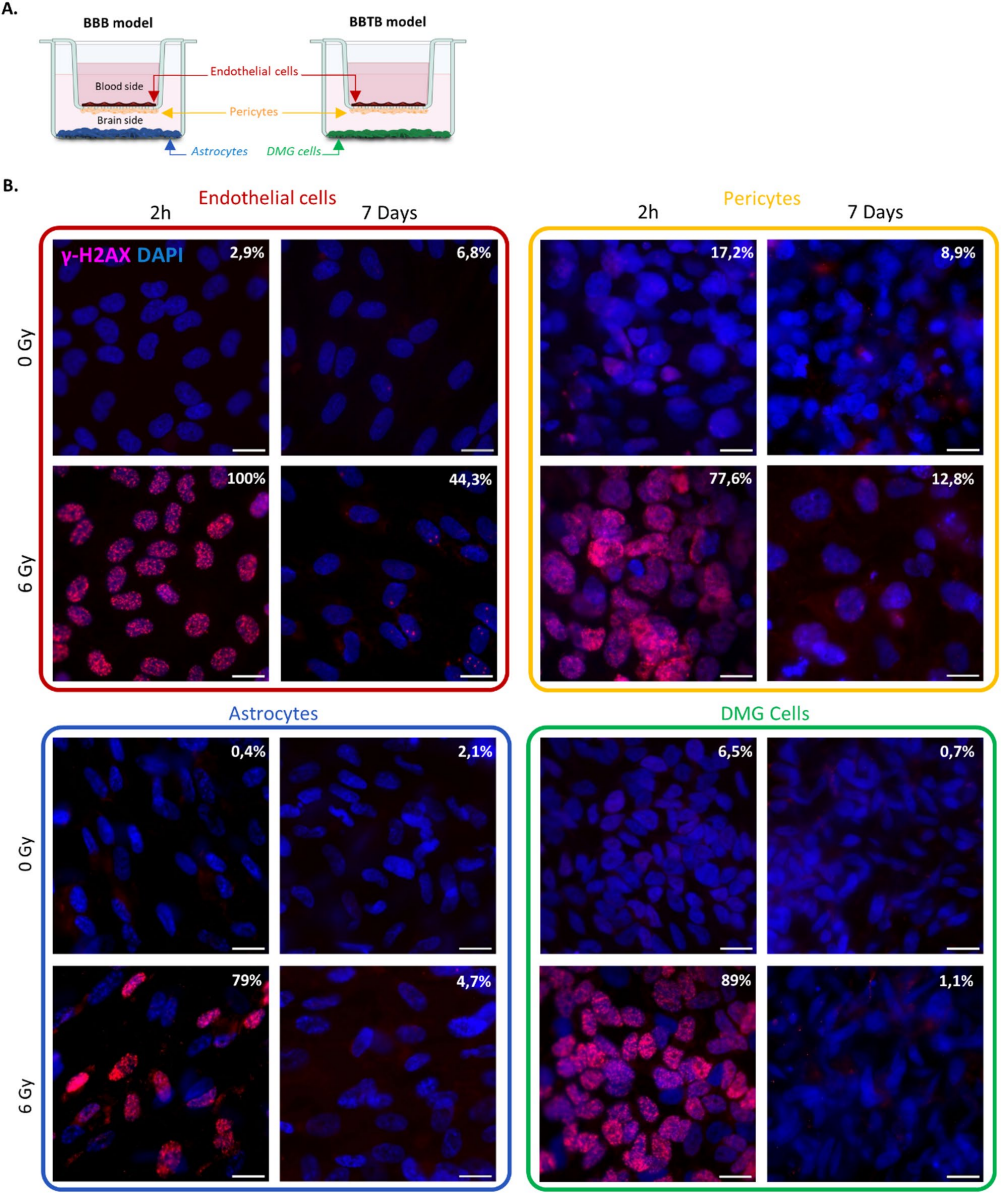
Induction of double-strand breaks in BBB/BBTB models. (**A**) Setting of Human Syngeneic BBB and BBTB in vitro models consisting of a triple culture of ECs, hBPs, and astrocytes (for BBB model) or DMG cells (for BBTB models). (**B**) Representative images of immunofluorescent staining of the phosphorylation of histone H2AX (γ-H2AX) to visualize the DSBs 2 h and 7 days after irradiation at 0 and 6 Gy in all cell types. The percentage represents the proportion of γ-H2AX-positive cells. Scale bar = 10 μm

To investigate how radiations, impact the BBB and BBTB properties, triple culture models consisting of ECs, hBPs and astrocytes for the BBB model or ECs, hBPs and DMG cells (HSJD-DIPG-007 or HSJD-DIPG-013) for the BBTB-007 and BBTB-013 models (Fig. 1A) were exposed to a single dose of radiation ranging from 2 Gγ to 6 Gγ. To ensure a uniform exposure of all cell types, DNA double-strand breaks were visualized at 2 h and 7 days post-irradiation using γ-H2AX staining. As shown in Fig. 1B (and in Additional file 1 A&B), representative images taken 2 h after single dose of 6 Gγ exposure, revealed a homogeneous distribution of γ-H2AX foci in the nuclei of all cell types. At 2 h post-irradiation, 100% of the ECs nuclei were positive (similar results were obtained for BBTB-007 ECs and BBTB-013 ECs, data not shown), 77.6% of hBPs nuclei, 79% of astrocytes and 89% of DMG nuclei. The non-individualization of γ-H2AX foci has rendered impossible the quantification of γ-H2AX foci per nucleus. However, the induction of DNA double-strand breaks seemed to increase with the dose. At 24 h post-irradiation, from the 4 Gγ dose, even if the number of γ-H2AX-positive nuclei remained the similar than at 2 h post-irradiation, the number of foci per nucleus has already decreased (Additional file 1).

At 7 days post-irradiation, the number of positive nuclei had dramatically declined. All cell types showed a partial or almost complete recovery, with 55.7% of ECs nuclei negative for γ-H2AX staining at 6Gγ, 87.2% of hBPs nuclei, 95.3% of astrocytes and 98.9% of DMG. Even if after 7 days, the endothelium does not present a complete recovery of DSB after exposure to 6 Gγ, for the 44.3% positive cells, the number of γ-H2AX positive foci per cell which is between 1 and 6 (mostly 2), is reduced compared to 2 h and 24 h conditions.

### Impact of radiation on BBB/ BBTB physical integrity

**Fig. 2:**
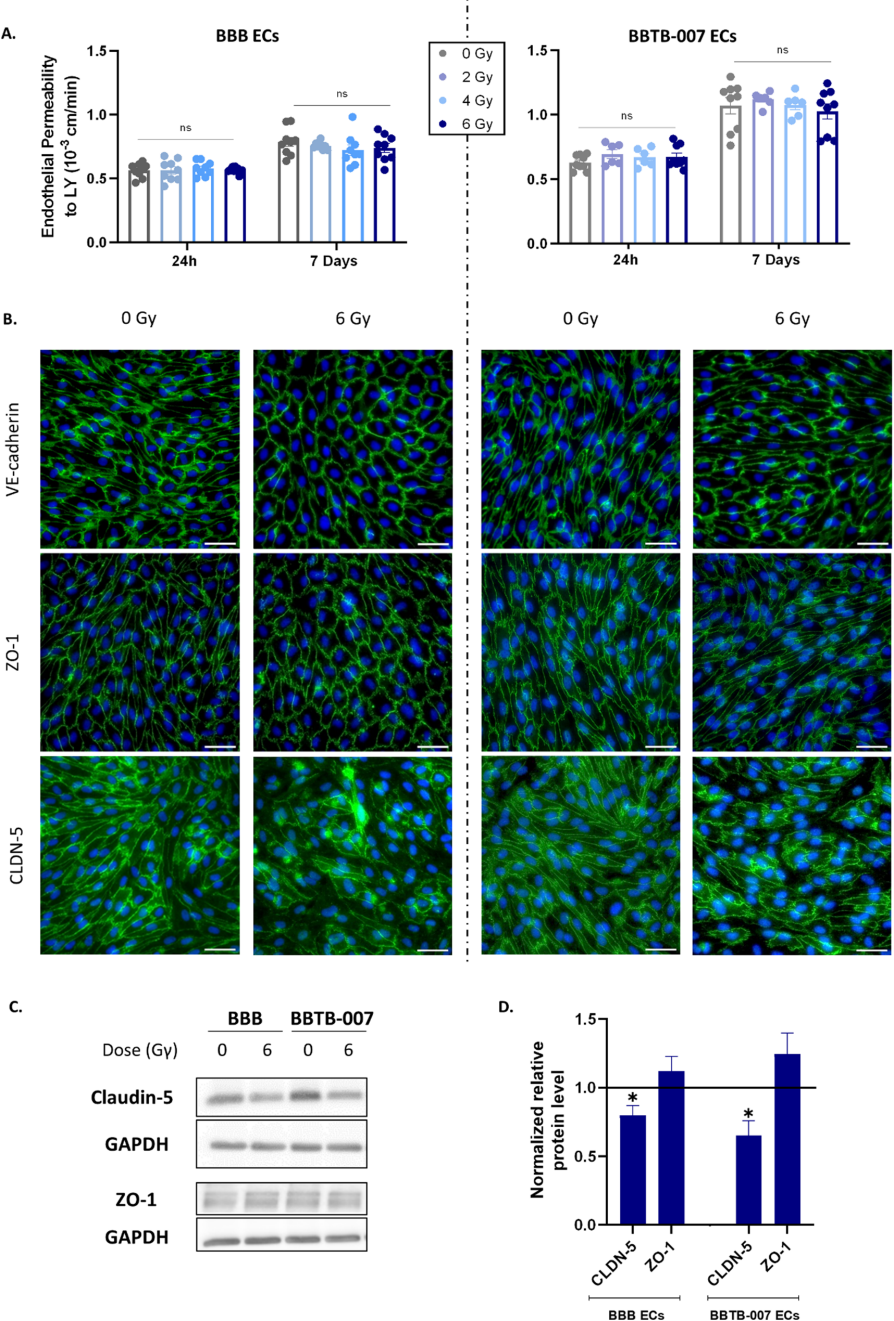
Investigation of the effects of irradiation on physical properties of the BBB and BBTB-007. (**A**) Endothelial permeability to Lucifer Yellow of BBB or BBTB-007 (DIPG-007 cells) models 24 h and 7 days after irradiation. Pe = endothelial permeability coefficient (10^− 3^ cm/min). One-way ANOVA followed by Dunnett’s post-hoc test, ns = non-significant, compared to the non-irradiated control, (BBB *N* = 3; *n* = 9, BBTB-007 *N* = 2; *n* = 6). A statistical analysis with “outliers” was performed in Additional File 7 A. (**B**) Representative images of adherent junctions VE-cadherin and tight-junction-associated proteins zonula occludens-1 (ZO-1), Claudin-5 (CLDN-5), 7 days after irradiation. Scale bar = 25 μm (**C**) & (**D**) Protein levels of Claudin-5 and ZO-1 in BBB and BBTB-007 ECs, 7 days after irradiation at a dose of 6 Gy, quantified by Western blot and normalized to GAPDH protein level. The line represents the non-irradiated control *p<0.05. (*N* = 3) Unpaired t-test was used for statistical study

Following the gradual single dose exposures, the physical integrity of the BBB ECs and BBTB ECs was assessed by measuring endothelial permeability to LY, a BBB integrity marker. A longitudinal follow-up was done for the permeability assay at 24 h and 7 days. As shown in Fig. 2A (left panel), 24 h post-irradiation, the permeability of the BBB ECs remained similar to the non-irradiated condition. Hence, after 2, 4 or 6 Gγ, the endothelial permeability was 0.56 ± 0.027 × 10^− 3^ cm/min, 0.58 ± 0.019 × 10^− 3^ cm/min and 0.57 ± 0.008 × 10^− 3^ cm/min respectively compared to the non-irradiated control condition (0.56 ± 0.018 × 10^− 3^ cm/min). At 7 days post-irradiation, no physical leakage of the BBB ECs monolayer was measured, since the endothelial permeability following 2, 4 or 6 Gγ was 0.76 ± 0.01 × 10^− 3^ cm/min, 0.72 ± 0.04 × 10^− 3^ cm/min, 0.74 ± 0.035 × 10^− 3^ cm/min respectively, compared to the non-irradiated control condition (0.79 ± 0.036 × 10^− 3^ cm/min).

The low endothelial permeability to LY was in line with the continuous localisation of the VE-cadherin, ZO-1 and CLDN-5 at the cell borders at 24 h post-irradiation for the BBB ECs (Additional file 2). At 7 days after irradiation, the expression of adherent junction VE-cadherin remains continuous at the endothelial cell borders, (Fig. 2B, Additional file 3). Concerning the tight junction proteins, whereas the expression of ZO-1 protein is also maintained and continuously localised at the endothelial cell borders, CLDN-5 displays a decrease in protein expression and a visible heterogeneous localisation in ECs after 6 Gγ radiation exposure (Fig. 2B, C&D).

The physical integrity of the BBTB-007 ECs was also maintained 24 h after irradiation compared to the non-irradiated control, 24 h post-irradiation the endothelial permeability following 2, 4 or 6 Gγ were 0.69 ± 0.034 × 10^− 3^ cm/min, 0.67 ± 0.028 × 10^− 3^ cm/min and 0.67 ± 0.029 × 10^− 3^ cm/min respectively, compared to the non-irradiated control (0.62 ± 0.019 × 10^− 3^ cm/min). (Fig. 2A right panel). Seven days after irradiation, the endothelial permeability to LY of the non-irradiated control of the BBTB-007 ECs was slightly higher compared to the value measured at 24 h. However, the endothelial permeability values at 2, 4, and 6 Gγ were 1.12 ± 0.023 × 10⁻³ cm/min, 1.08 ± 0.037 × 10⁻³ cm/min, and 1.02 ± 0.058 × 10⁻³ cm/min, respectively, were not statistically different compared to the non-irradiated control (1.07 ± 0.063 × 10⁻³ cm/min). Continuous localisation of adherent junctions VE-cadherin and tight junction proteins ZO-1 was observed in the BBTB-007 ECs monolayer from 24 h to 7 days post-irradiation (Fig. 2B right panel, Additional file 2 & 3). As observed in the BBB ECs, CLDN-5 expression was also decreased 7 days after 6 Gγ of irradiation in the BBTB-007 ECs and displayed a heterogeneous pattern of localisation at the ECs borders (Fig. 2B).

The quantification by western blot of the tight junction proteins confirmed the observations made from immunostaining images since the expression of ZO-1 was maintained in BBB and BBTB ECs following radiation. However, irradiation induced a significant decrease of 21% and 35% in CLDN-5 expression in the BBB and BBTB ECs, respectively after 7 days (Fig. 2C&D).

The results obtained for the BBTB-013 ECs, were similar to BBB ECs with a preservation of ECs integrity in terms of junctions and endothelial permeability to LY (Additional file 2, 3 and 4). Following 2, 4 or 6 Gγ, the endothelial permeability values were 0.59 ± 0.027 × 10^− 3^ cm/min, 0.59 ± 0.029 × 10^− 3^ cm/min and 0.62 ± 0.032 × 10^− 3^ cm/min respectively compared to the non-irradiated control (0.60 ± 0.029 × 10^− 3^ cm/min). At 7 days post-irradiation, the endothelial permeability values were 0.71 ± 0.08 × 10^− 3^ cm/min, 0.66 ± 0.05 × 10^− 3^cm/min, 0.63 ± 0.054 × 10^− 3^ cm/min respectively for 2, 4 or 6 Gγ, compared to the non-irradiated control condition (0.71 ± 0.067 × 10^− 3^ cm/min). Contrary to the BBTB-007 ECs, the BBTB-013 ECs displayed a preserved homogeneous localisation of adherent and tight junction proteins associated with a maintenance of levels expression after radiation (Additional file 4).

However, despite the preserved endothelial permeability at 7 days for the three models, a focus on the ECs count has revealed a reduction in cell number. This decrease was significant at 2 Gγ for the BBB model and at 4 Gγ for the BBTB-007 model and BBTB-013 (Additional file 5 A). For the 6 Gγ condition, a decrease of 16.5%, 14.4% and 18.5% were observed for BBB, BBTB-007 and BBTB-013 respectively.

In addition, a significant decrease in the metabolic activity of BBB and BBTB-007 ECs was also measured at 7 days after 6 Gγ of irradiation, with a respective decrease of 15% and 22% compared to the non-irradiated control and 9% for BBTB-013 ECs (Additional file 5B).

### Consequences of radiation on BBB & BBTB at the molecular level: focus on the metabolic properties

Metabolic properties were analysed at a molecular level to further understand the consequences of irradiation on the BBB/BBTB ECs. To do so, gene expression analysis was performed on ECs and hBPs and focused on selected efflux pumps and detoxification enzymes known to be involved in the chemoresistance mechanisms by modulating drug bioavailability. The analysed efflux pumps were P-gp, BCRP, MRP1, MRP2, MRP4 and MRP5 (respectively genes names *ABCB1*,* ABCG2*,* ABCC1*,* ABCC2*,* ABCC4* and *ABCC5*) and the detoxification enzymes from the cytochrome P450 family (CYPs) were *CYP1A1*,* CYP1B1*,* CYP2D6 and CYP2S1*.

**Fig. 3:**
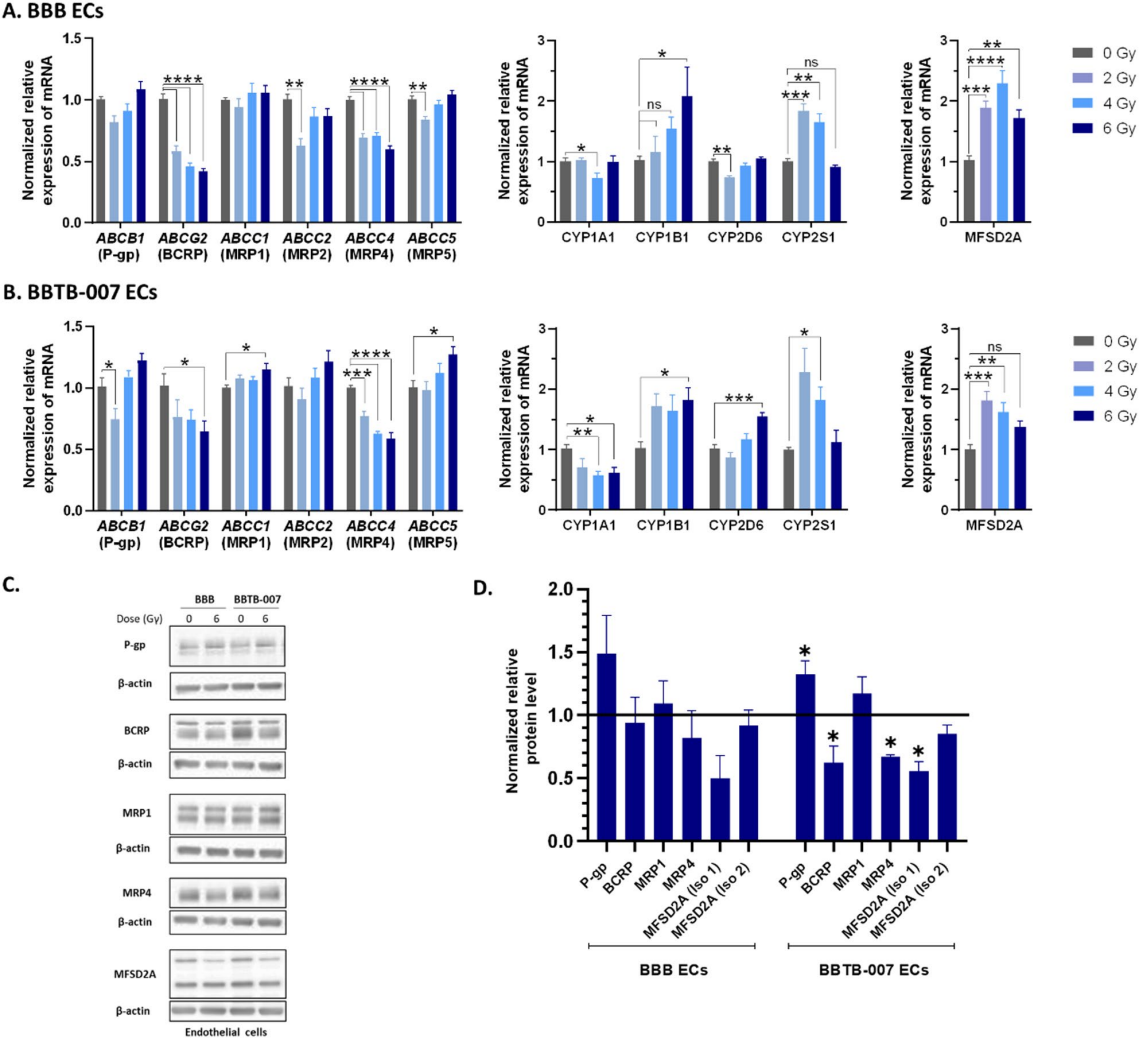
Characterization of metabolic properties of BBB ECs and BBTB-007 ECs after irradiation. Transcriptional ECs genes expression analysis for efflux transporters (P-gp, BCRP, MRP1, MRP2, MRP4, MRP5), detoxification enzymes (cytochrome P450 enzymes CYP1A1, CYP1B1, CYP2D6, CYP2S1), and transporter MFSD2A, 7 days after irradiation of 2, 4 and 6 Gγ in BBB ECs (**A**) and BBTB-007 ECs (**B**). The gene expression was quantified by RT-qPCR and normalized on the expression of the housekeeping gene GAPDH. For the statistical study One-way ANOVA followed by Dunnett’s multiple comparisons test was used ns = non-significant *p < 0.05, **p < 0.01, ***p < 0.001, ****p < 0.0001, compared to the non-irradiated control (BBB *N* = 3; *n* = 9, BBTB-007 *N* = 2; *n* = 6). (**C**) & (**D**). Protein levels of efflux transporters P-gp, BCRP, MRP1, MRP4 and MFSD2A (Isoforms (Iso) 1&2), 7 days after irradiation at a dose of 6 Gγ were quantified by Western blot and normalized to protein level of β-actin. The line represents the level of expression in non-irradiated control. Unpaired t-test was used for statistical study, (*N* = 3)

As shown in Figs. 3A and 7 days after irradiation, no modulation of mRNA level was measured for *ABCB1* and *ABCC1* in BBB ECs. However, significant reductions of mRNA expression of *ABCG2* and *ABCC4* were quantified in BBB ECs, with a decrease of 58% and 41% respectively at 6 Gγ compared to the non-irradiated control. Hence, similarly to BBB ECs, the BBTB-007 ECs displayed a decrease in *ABCG2* and *ABCC4* with a decrease of 36% and 41% respectively induced by 6 Gγ irradiation. In addition, there was an increase in *ABCC1* and *ABCC5* mRNA levels (Fig. 3B). Similar results to BBB ECs were obtained for BBTB-013 ECs (Additional file 6 A).

At the protein level, whereas no significant modulation of MRP1 expression was measured in the BBB and BBTB ECs induced by irradiation (Fig. 3C&D and Additional file 6 C&D). A significant increase in P-gp was measured only in the BBTB-007 ECs. In addition, a significant decrease in BCRP and MRP4 expression was measured following irradiation, with levels reducing by 38% for BBTB-007 ECs and 38.2% for BBTB-013 ECs; and by 34% for BBTB-007 ECs and 32% for BBTB-013 ECs respectively.

**Fig. 4:**
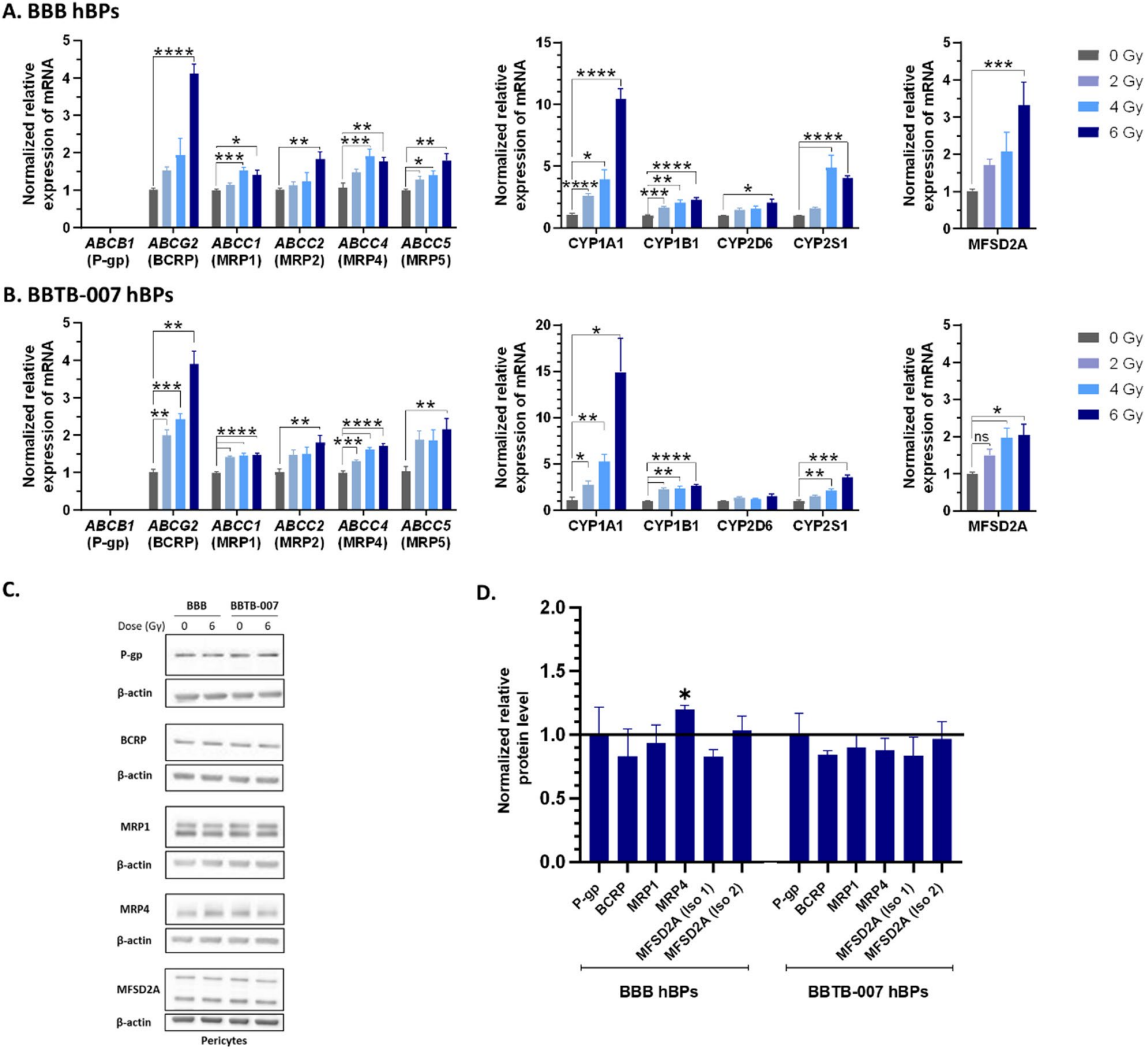
Characterization of metabolic properties on hBPs of BBB and BBTB-007 after irradiation. Transcriptional hBPs genes expression for efflux transporters (P-gp, BCRP, MRP1, MRP2, MRP4, MRP5), detoxification enzymes (cytochrome P450 enzymes CYP1A1, CYP1B1, CYP2D6, CYP2S1), and transporter MFSD2A, 7 days after irradiation of 2, 4 and 6 Gγ in BBB hBPs (**A**) and BBTB-007 hBPs (**B**). The gene expression was quantified by RT-qPCR and normalized on the expression of the housekeeping gene GAPDH. For the statistical study One-way ANOVA followed by Dunnett’s multiple comparisons test was used, ns = non-significant *p < 0.05, **p < 0.01, ***p < 0.001, ****p < 0.0001, compared to the non-irradiated control. (BBB *N* = 3; *n* = 9, BBTB-007 *N* = 2; *n* = 6). (**C**) & (**D**). Protein levels of efflux transporters P-gp, BCRP, MRP1, MRP4 and MFSD2A (Isoforms (Iso) 1&2), 7 days after irradiation at a dose of 6 Gγ, quantified by Western blot and normalized to protein level of β-actin. The line represents the level of expression in non-irradiated control. Unpaired t-test was used for statistical study, (*N* = 3)

The same analyses were performed in parallel, in BBB and BBTB hBPs, 7 days post-irradiation. Expression of *ABCB1* was not detected in hBPs. For the other targets of the *ABCC* family and also for *ABCG2*, the mRNA expression followed a dose-dependent profile and was significantly increased especially for *ABCG2* reaching a 4-fold increase after 6 Gγ of irradiation (Figs. 4A&B and Additional file 6 A&B). At the protein level, 7 days after 6 Gγ of irradiation, no significant modulation was observed for P-gp, BCRP, and MRP1. However, MRP4 is increased in BBB hBPs. (Figs. 4C&D and Additional file 6 C&D).

Concerning the detoxification enzymes, despite the measurement of a transitory increase for the four cytochromes P450 tested on BBB ECs, only *CYP1B1* displayed a 108% increase at 6 Gγ in BBB ECs (Fig. 3A).

In the BBTB ECs condition, the expression of *CYP1B1* was increased by 82% and there was also a significant increase in *CYP2D6* expression for BBTB-007 ECs (Fig. 3B) and an increase of 217% for *CYP1B1* in BBTB-013 ECs (Additional file 6 A). In addition, although the *CYP2S1* presented an increase in expression at 2 and 4 Gγ, this was not measured at 6 Gγ for BBB ECs, BBTB-007 ECs and BBTB-013 ECs (Figs. 3A&B and Additional file 5 A).

In hBPs, similar results were observed for the 4 CYPs tested, with a particularly significant increase for *CYP1A1*, whose mRNA expression exhibited a 10.45; 14.9 and 12.8-fold increase for BBB; BBTB-007 and BBTB-013 respectively after a dose of 6 Gγ. (Figs. 4A&B and Additional file 6B).

In addition to the study of efflux pumps and detoxification enzymes, we then focused on MFSD2A, a protein known as a transporter of DHA (DocosaHexaenoic Acid) and involved in BBB restrictive properties establishment. Hence, the expression of MFSD2A was investigated in BBB and BBTB ECs and hBPs following irradiation. A significant increase in mRNA expression was measured for BBB ECs (Fig. 3A), but it remained not significant for BBTB ECs after 6 Gγ (Fig. 3B and Additional file 6 A). In hBPs, *mfsd2a* mRNA expression exhibited a significant 3.3-fold, 2.4-fold and 1.9-fold increase for BBB, BBTB-007 and BBTB-013 respectively (Figs. 4A&B and Additional file 6B). At the protein level, while the isoform 1 of MFSD2A exhibited a decrease of 50% of expression in the BBB, 44% in BBTB-007 ECs and 53% on BBTB-013 ECs (Fig. 3C&D, Additional file 6 C&D), the expression of the isoform 2 remained unchanged compared to the non-irradiated condition. In the hBPs, both isoforms expression remained unchanged in comparison to the non-irradiated condition (Fig. 4 C&D, Additional file 6 C&D).

In addition, since irradiation induced a decrease in *BCRP* expression at mRNA and protein levels in ECs but maintained its expression in hBPs, functionality tests were performed.

To do so, the P-gp / BCRP activity was evaluated in ECs after irradiation by measuring intracellular accumulation of Rhodamine 123, in the presence or absence of Elacridar, an inhibitor of P-gp / BCRP.

**Fig. 5:**
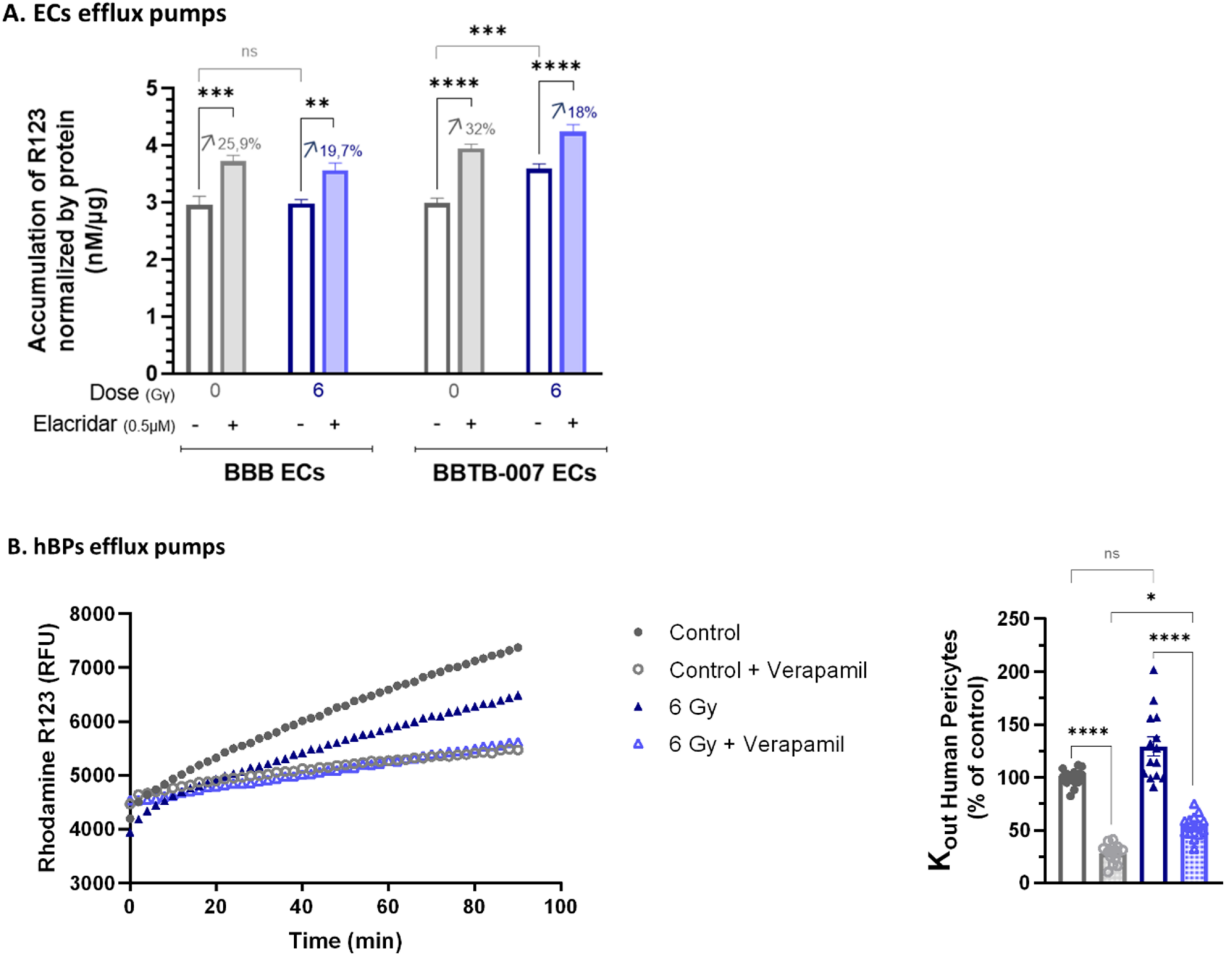
Investigation of P-gp / BCRP activity in ECs and hBPs of BBB / BBTB-007 models. The analyse of efflux pumps activity was performed 7 days after 6 Gγ exposure. (**A**) The activity of P-gp / BCRP efflux pumps is evaluated by accumulation of P-gp / BCRP substrate rhodamine 123 (R123) in ECs after incubation with or without 0.5µM of inhibitor, elacridar. This histogram represents the concentration of R123 accumulated in ECs per µg of protein. The percentages indicate the increase in accumulation between the control and the condition with inhibitor for each group. One-way ANOVA followed by Tukey’s multiple comparisons test was used for the statistical study (*N* = 2; *n* = 8). (**B**) The activity of P-gp / BCRP efflux pumps is evaluated by efflux of rhodamine 123 from hBPs after incubation with or without 50µM of inhibitor, verapamil. The kinetic is represented on the left panel) and excretion rate of R123 on the right panel. The results were represented as a percentage relative to the control (100% = 32.2 dRFU/dt/ng of proteins). The Kruskal-Wallis test followed by Dunn’s multiple comparisons test was used for the interpretation of statistical data. ns = non-significant, *p < 0.05, **p < 0.01, ***p < 0.001, ****p < 0.0001, (*N* = 3; *n* = 14)

In BBB ECs, the intracellular accumulation of rhodamine R123 remained unchanged between non-irradiated and irradiated BBB ECs (2.96 and 2.98 nM/µg, respectively). The presence of elacridar induced an increase in intracellular accumulation of R123 by 25.9% (3.73 nM/µg), revealing an active efflux transport. After irradiation, this increase in accumulation induced by the inhibitor was reduced to 19.7% (Fig. 5A). Although the impact of Elacridar on intracellular R123 accumulation was less after irradiation, suggesting a decrease of efflux efficiency, it remained non-significant compared to the non-irradiated control.

Concerning the BBTB-007 ECs, in absence of inhibitor, a significant increase in R123 accumulation was measured following radiation compared to the non-irradiated control (3.59 nM/µg and 2.99 nM/µg respectively). Whereas a 32% increase of intracellular accumulation was measured in the non-irradiated control treated with elacridar, the inhibitory effect of elacridar was reduced to 18% after irradiation. Taking together, these results suggest a decrease of efflux pump efficiency in BBTB-ECs after irradiation.

Concerning the hBPs, since these cells were seeded in the reverse side of the insert for the BBB and the BBTB models, the evaluation of P-gp / BCRP activity after irradiation cannot be done using the same experimental procedure. For this reason, the co-culture model consisting of ECs and hBPs was used for this functionality test. The P-gp / BCRP assay was performed using a pump-out assay, where the efflux of rhodamine was monitored in real-time in the presence or absence of inhibitor.

As presented in Fig. 5B, the kinetics of R123 efflux were plotted versus time. Hence, in the presence of Verapamil (Empty dots), a P-gp / BCRP inhibitor, the slope of R123 release was reduced compared to the control without inhibitor (full dot). The slower rate of clearance of R123 measured in presence of Verapamil in irradiated and non-irradiated hBPs indicate the functionality of P-gp/ BCRP in these cells. The calculated K_out_ of R123, representing the rate of clearance of R123 from the hBPs was reduced in presence of Verapamil by 72.59% and 59,32% in non-irradiated or irradiated hBPs respectively (Fig. 5B, right panel). Hence irradiation induced a significant decrease in P-gp / BCRP activity in hBPs.

### Inflammation-induced damages are exacerbated in BBB ECs after irradiation but not in BBTB ECs

To mimic the release of pro-inflammatory cytokines induced in irradiation, the in vitro models were treated with TNFα, a key pro-inflammatory mediator. To do so, the TNFα was added in the cerebral compartment of the cellular system to simulate cytokines release from the brain parenchyma.

**Fig. 6:**
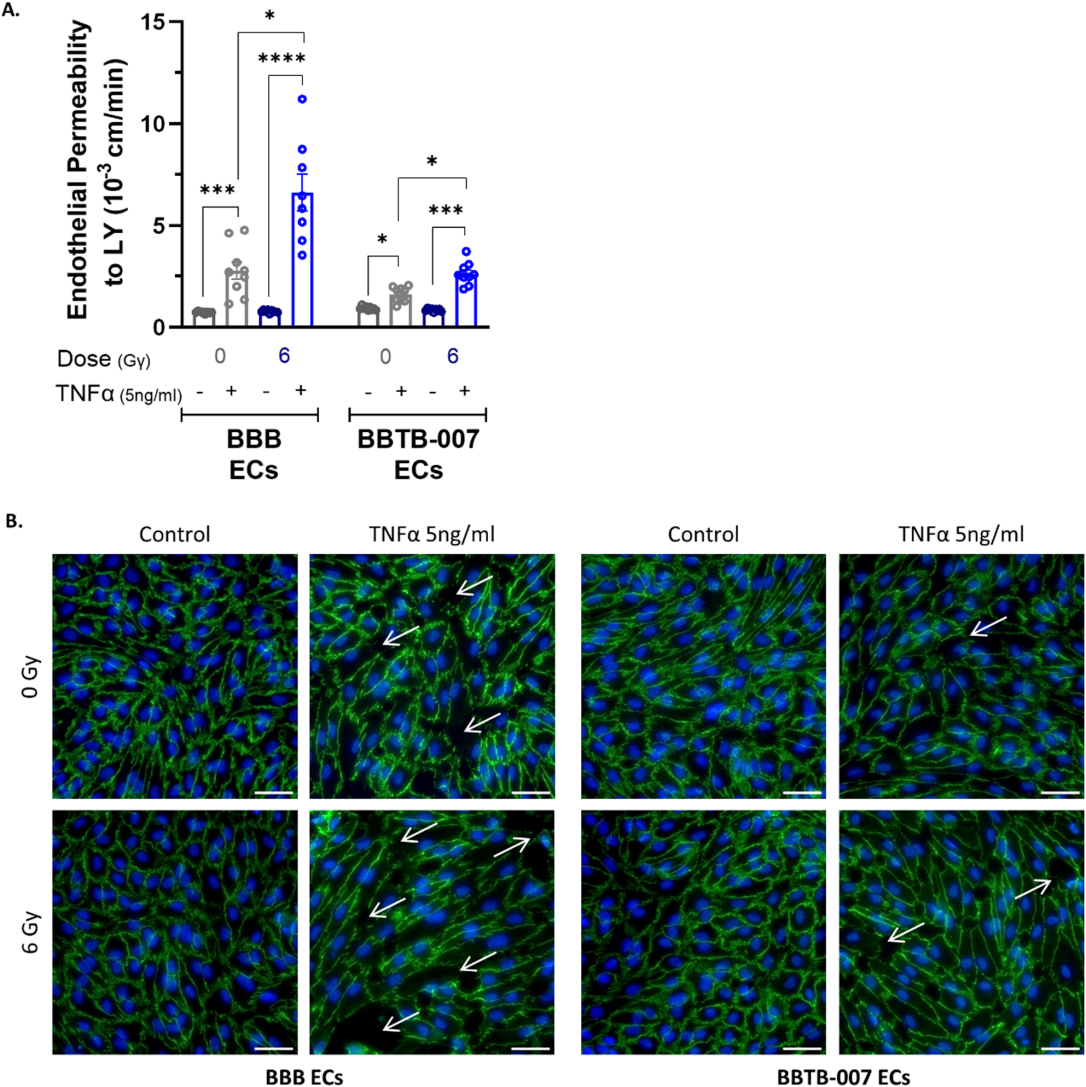
Effect of irradiation combined with TNFα-mediated inflammation. To mimic an inflammatory exposure, 24 h after irradiation, the BBB and BBTB-007 models were treated in the cerebral compartment with 5 ng/ml TNFα, for 24 h. The physical properties of the barriers were evaluated. (**A**) Permeability of endothelial monolayer to Lucifer Yellow. For the statistical study One-way ANOVA followed by Dunnett’s multiple comparisons test was used. *p < 0.05, ***p < 0.001, ****p < 0.0001 (*N* = 3; *n* = 9) (**B**) Representative images of tight-junction-associated protein ZO-1 expression. White arrows indicate loss of junctions between cells. Scale bar = 25 μm

After 24 h of TNFα exposure, the BBB ECs exhibited a significant 3-fold increase in endothelial permeability compared to the non-treated condition (2.77 ± 0.42 × 10^− 3^ cm/min and 0.73 ± 0.02 × 10^− 3^ cm/min respectively). Notably, when the BBB ECs were irradiated, the increase in endothelial permeability was 8.6-fold higher relative to the untreated condition (6.62 ± 0.89 × 10^− 3^ cm/min and 0.77 ± 0.02 × 10^− 3^ cm/min respectively) (Fig. 6A). These endothelial permeability changes induced by TNFα were correlated with immunostaining of ZO-1 (Fig. 6B left panel), where visible interruptions of the staining were observed, as indicated by white arrows, with a higher frequency when combined with irradiation.

Interestingly, the impact of TNFα treatment on the BBTB-007 ECs was less important than on the BBB-ECs. Indeed, in the non-irradiated condition, following the treatment with TNFα, the increase in endothelial permeability was 1.7-fold higher relative to the untreated condition of the BBTB ECs (1.6 ± 0.13 × 10^− 3^ cm/min and 0.92 ± 0.03 × 10^− 3^ cm/min respectively). However, after irradiation, TNFα treatment resulted in a 3.1-fold increase in endothelial permeability compared to the non-treated condition (2.62 ± 0.19 × 10^− 3^cm/min and 0.84 ± 0.02 × 10^− 3^ cm/min respectively). These results were correlated with the staining of ZO-1 revealing some interruptions of the tight junctions within the monolayer (Fig. 6B right panel).

## Discussion

Recent advances in the characterization of DMG, along with the integration of molecular features, have emerged since the new classification of brain tumors in 2016 [31]. These developments highlight the complexity of brain tumors and underscore the importance of studying each tumor type individually to enable the development of personalized therapies. Nevertheless, despite advances in understanding the mechanisms underlying treatment responses at the tumor cell level, treatment resistance remains challenging and represents a major obstacle [32]. This is why, radiotherapy still remains the only standard of care for DMG. Therefore, considering not only the tumor cells but also the complexity of the TME is prime of importance [33]. Indeed, while the role of the TME in tumor progression is now widely accepted, characterising the TME in treatment response remains to be explored as an actor in the modulation of the treatment efficiency. The TME of DMG is a complex environment that encompasses multiple cell types in addition to tumor cells, extracellular matrix as the acellular component but also the vasculature. The brain tumor vasculature is composed of brain capillaries responsible for nutrient and oxygen supply but, also limiting the treatment efficacy [34, 35]. Indeed, the specific and restrictive properties of the brain capillaries include physical and metabolic properties that are regulated by communications among the NVU. Moreover, the presence of cancer cells interferes with these communications and plays a pivotal role in the regulation of the BBB properties which is then renamed BBTB. The physical and metabolic characteristics of the BBTB of DMG are still poorly understood. The development of relevant preclinical models that combine both tumor biology and the TME is prime of importance for understanding the treatment responses and also for advancing effective treatments. For this reason, we have previously developed a BBTB model specific to the DMG [21, 24].

Our experimental in vitro approach uses a syngeneic human BBB model consisting in ECs cultivated with hBPs and astrocytes [21, 24]. This triple culture model has been adapted from a patented co-culture model [25]. Moreover, in order to model specifically the vasculature of the DMG, a BBTB model has been developed by including DMG cells instead of astrocytes [21]. The setting of these models allowed to integrate the reciprocity of cell-cell communication in the induction of BBB phenotype but also following disease or treatment conditions and at the same time to separately collect each cellular component for further molecular analysis. Our previous results have demonstrated that unlike other brain tumors, DMG cells don’t lead to physical disruption of the BBTB [21], as already suggested by clinical and in vivo results [20, 10]. Although DMG tumor displays a strong chemoresistance, our previous study has demonstrated that the metabolic BBTB properties were not strengthened [21]. Hence, in the present study using the BBTB model, we aimed at studying the impact of radiotherapy on the tumor vasculature by focusing on physical and metabolic properties involved in drug delivery.

The model has been exposed to a single dose of radiation, ranging from 2 Gy to 6 Gγ, using a contact device. According to the literature, doses used comprised a range of radiations commonly used in cell culture systems, starting at 2 Gγ, which represents the reference dose in clinic for patients that receive a fractionated radiotherapy protocol and allows to assess the consequences of radiation by inducing measurable effects without massive cell death occuring at early stage post-irradiation [36–38]. In fact, the doses tested in an in vitro model do not correspond to the total doses received by patients during treatment protocol, but rather to a cumulative dose administered at the beginning of treatment after one (2 Gy), two (4 Gy), and three (6 Gy) radiotherapy sessions, in order to understand how BBTB can be modulated at the beginning of the therapeutic session. In addition, the dose of 6 Gγ is close to the estimated dose of healthy tissue exposed to a single exposure (7 Gγ) [39]. Anyhow, the different doses were all able to induce significant DNA damage in all cell types of the cell culture system since the nuclei of all cells had displayed a positive staining for the DSB. In addition, even if the density of DSB foci in nuclei has rendered impossible the quantification, the quantity of DSB foci increased with the dose received. This effect, was also observed at 7 days since the foci were still detected in some nuclei for the dose 6 Gγ but not for the 2 Gγ. In addition, this dose response effect was also observed on endothelial cell count, since a significant decrease in endothelial cell number was measured after 6 Gγ in the BBB and the BBTB. These results are in line with a previous study using a primary bovine in vitro BBB model where the follow-up has been done until 7 days post-irradiation [37]. However, following radiation, cell usually undergoes cell death; our study showed that the decrease in cell number was not correlated with a loss of endothelium physical integrity whatever the dose of radiation. Nevertheless, a significant decrease in the metabolic activity of endothelial cells was observed 7 days after exposure to 6 Gy of radiation. The intercellular spaces between the endothelial cells of the BBB are sealed by tight junctions, which help maintain the physical integrity of the BBB. The permeability of the endothelium after irradiation remains similar to the non-irradiated control and correlates with the staining of VE-cadherin adherent junctions and ZO-1 tight junctions. Claudin-5 is one of the main tight junction proteins expressed by ECs, is responsible of the restriction of paracellular permeability to small molecules (< 800Da) as described in Claudin-5 knock-out mice [40]. In our study, Claudin-5 expression in ECs appears to be heterogeneous after irradiation. Our results were similar in the BBB and BBTB ECs, but do not fully correlate with other in vitro studies using animal models. In these studies, the BHE showed a significant increase in permeability one week after irradiation with 2 Gγ and 4 Gγ [37, 41, 42]. However, these models used ECs co-cultured with mixed glial cells and did not include hBP. In the literature, the question of BBB/BBTB dysfunction or leakage after irradiation is still debated [43, 44] The physical integrity of the BBB/BBTB depends on the radiation dose, follow-up kinetics, and experimental model used in preclinical studies. Nevertheless, radiation-induced damage to the BHE is often associated with inflammatory events [45].

In fact, radiotherapy is known to induce inflammation, which increases tissue damage severity [46, 47] Following radiotherapy, patients receive anti-inflammatory treatment with corticosteroids in order to limit oedema formation [10, 48]. Hence, in vitro studies, demonstrating radiation-induced BBB damages, have used cellular models containing microglial cells, known to be the resident immune cells in the central nervous system, alone or within a mix of glial cells population [37, 45, 47]. The absence of microglia cells in our cell culture model could explain the maintenance of the physical integrity. Astrocytes and DMG cells are not sufficient to create a pro-inflammatory condition following radiation in the time frame of our study. Nevertheless, when the release of pro-inflammatory cytokines was mimicked by a TNFα treatment, a disruption of the BBB was measured and demonstrates that irradiation exacerbates by 8.6-fold the increase in the endothelial damages induced by inflammatory stress [49]. Interestingly, this effect was measured in a much lesser extent in the BBTB model, since after irradiation, TNFα treatment induced a 3.1-fold increase in permeability, revealing that the tumoral DMG environment trends to maintain the endothelium integrity. This result is in correlation with the clinical situation since, contrary to other types of brain tumor such as glioblastoma, DMG is known to be a « cold » tumor, with a specific DMG TME where at any stage there is no major lymphocyte infiltration [50–52] and in line with a recent in vitro data where the DMG were demonstrated to maintain an immunosuppressive environment [53]. Indeed, irradiation does not provoke only DNA damages; its effects extend more broadly to the entire cell components, and can impact the plasma membrane [54, 55]. This could explain the potentiating effect of TNFα exposure after irradiation on the BBB ECs [55]. In our study, using TNFα allowed to mimic the neuro-inflammation stage following radiation. This stage occurs as a consequence of radiation-induced pro-inflammatory cytokines release [56–58]. As one of the major pro-inflammatory cytokines released during the neuro-inflammation, TNFα is widely used in experimental setting in vitro [27, 59]. In addition, the permeation effect of TNFα is now tested as a therapeutic treatment for brain tumors. In fact, the combination of chemotherapeutic drug with TNFα derivatives is now developed to selectively increase the BBTB permeability to improve the drug delivery [60]. This approach is particularly useful because it allows for the permeabilization of the BBB/BBTB in tumors with an intact BBB physical integrity, as is the case with DMG. To further study radiation-induced neuro-inflammation in our BBTB model and also take into account intratumoral heterogeneity, DMG cells could be replaced by organoids derived from surgical resection [61, 62]. However, the complex location of DMG and the rarity of samples make it difficult to collect representative tumor samples that accurately reflect the intratumoral heterogeneity of this tumor.

Over the past few decades, clinical trials have been tested the combination of radiotherapy and chemotherapy against DMG to improve the therapeutic strategies However, the chemotherapeutic or immunotherapy agents tested are generally substrates of efflux pumps (P-gp, BCRP, MRP1) which limits their access to the tumor cells [19, 63]. Therefore, studying the effects of radiation on efflux pumps expression and activity is important.

At a molecular level, focusing on the metabolic properties of the BBB and the BBTB reveals that at 7 days post-irradiation, expression of numerous efflux pumps and detoxification enzymes was modulated. For ECs, the efflux pumps BCRP and MRP4 expression was decreased in the BBTB following radiation. Moreover, the impact of radiation was more important in the BBTB-ECs since significant decrease in expression and activity of P-gp/ BCRP were measured. Further analysis is needed to identify the mechanisms responsible for changes in BCRP expression and activity. It cannot be ruled out that these results are the consequence of post-translational modifications and/or an increase in degradation pathways. In addition, the BBTB ECs are distinguished from the BBB ECs by an upregulation of MRP1 and MRP5. These two multidrug resistance proteins are described in the resistance to treatment of numerous brain cancers [64] and their upregulation was found in ependymoma and glioma and can represent potential targets to overcome the BBTB [64].

Interestingly, hBPs showed a compensatory effect, while BCRP expression was decreased in the endothelium, it was conversely maintained in hBPs. However, even reduced following irradiation, the activity of efflux pump P-gp/BCRP in hBPs remained efficient and still represents a mechanism to overcome to improve the drug delivery. In addition, no differences were measured from hBPs in the BBB and in the BBTB after irradiation.

Concerning the detoxification enzymes, the CYPs are able to metabolize many endogenous and exogenous compounds (such as chemotherapeutic drugs). In addition, certain CYPs are active in cerebral microvessels, such as CYP1A1, CYP1B1, CYP2D6, and CYP2S1, which are located in the BBB ECs [65, 66]. While transporters tend to influence the distribution of drugs, CYPs also impact their effect through biotransformation.

While BBB ECs show only an increase in *CYP1B1*, the BBTB ECs exhibits an increases in both *CYP1B1* and *CYP2D6*.

Consistent with the analysis of efflux transporters, irradiation induced an increase of CYPs expression in hBPs. Nonetheless, no differences were detected between hBPs from the BBB model and those from the BBTB. However, the time window of analysis may need to be extended for protein analysis since, even if the higher modulation rate was measured at 7 days post-irradiation for mRNA, for the majority of molecular target candidates no increase or decrease in proteins expression were validated in the BBB and BBTB ECs at 7 days. MRP4 and BCRP were the only ones to demonstrate a correlation between mRNA and protein expression only in BBTB ECs. Member of the multidrug resistance family, further investigations are required to understand the role of MRP4 in BBTB ECs.

Interestingly, MFSD2A, a lipid transporter known to be a marker of BBB establishment, has exhibited an increase in expression of mRNA transcript following radiation in all cell types [67]. However, its expression at protein level has demonstrated a regulated mechanism where only the first isoform was decreased. While the BBB/BBTB ECs physical integrity was maintained the decrease in protein expression can be a preliminary event that precedes BBB/BBTB disruption.

According to the literature, the maintenance of ECs physical integrity following irradiation can be linked to CYP1B1 and MFSD2A upregulation. Indeed, the metabolic activity of CYP1B1 was demonstrated to be involved in the maintenance of junction integrity and P-gp function. Moreover, its inhibition is associated with BBB leakiness [68]. Concerning MFSD2A, known to mediate uptake of the essential fatty acid DHA across the BBB, this protein is associated with BBB establishment and is strongly regulated by hBPs [69, 70]. Notably, reduced MFSD2A expression has been linked to BBB disruption in brain cancer [71]. Consequently, lipid metabolism emerges as a significant pathway warranting further investigation to enhance our understanding of the mechanisms underlying BBB/BBTB damages following radiation exposure. In addition, experimental data suggest also a link between MFSD2A and Claudin-5 expression [72, 73]. Additional experiments need to be conducted to decipher if a regulation pathway is existing.

Pericytes play a crucial role in the induction and regulation of the BBB ECs, but, at the molecular level, the characterization of pericytes is often overlooked in favour of ECs. Importantly, this regulatory mechanism highlights that regardless of the BBB’s physical integrity, pericytes contribute to treatment resistance, particularly in restricting the brain penetration of therapeutic agents, should not be underestimated. This may provide insight into the persistence of drug resistance in some cancer types even where structural integrity of the BBTB is compromised.

## Conclusions

In this study, we have demonstrated that in the absence of inflammatory stress, the physical integrity of the BBB and the BBTB was preserved but associated with heterogeneous distribution of Claudin-5 at the ECs borders and decreased of expression after irradiation. Moreover, the response to inflammation was one of the most relevant effect to distinguish the BBB from the BBTB. For metabolic properties, the compensatory effect observed with pericytes has clearly indicated the importance of pericytes in the regulation of BBB/BBTB properties. Additional molecular analyses are required to better characterize the consequences of irradiation on the BBTB ECs involving the fatty acid metabolism and transport, especially following inflammatory conditions. An update of the BBB model can be considered, instead of using pro-inflammatory cytokine, addition of microglial cells to the abluminal compartment of the cell culture system will represent a more physiological manner to study the post-irradiation inflammation.

## Supplementary Information

Below is the link to the electronic supplementary material.

Supplementary Material 1: Additional file 1. Induction of double-strand breaks (DSB) in BBB/ BBTB models. (A) Representative images of immunofluorescent staining of the phosphorylation of histone H2AX (γ-H2AX) to visualize the DSB at 2 h, 24 h and 7 days after irradiation at 0 to 6 Gγ in ECs and on hBPs at 2 h after irradiation dose at 0 to 6 Gγ. On images of ECs, the percentage of γ-H2AX positive cells is indicated. Scale bar = 10 µm. (B) Representative images of γ-H2AX to visualize the DSB at 2 h and 7 days after irradiation at 0 and 6 Gγ in human astrocytes brainstem, HSJD-DIPG-007 and HSJD-DIPG-013 present in the cerebral compartment of the model. Scale bar = 200 µm

Supplementary Material 2

Supplementary Material 3: Additional file 2. Study of the effects of irradiation on the expression of junctions in BBB/BBTB models. Representative images of adherent junctions VE-cadherin and tight-junction-associated proteins Zonula Occludens-1 (ZO-1), Claudin-5 (CLDN-5) 24 h after irradiation dose of 6 Gγ on the three models (BBB, BBTB-007 and BBTB-013). Scale bar = 25 µm

Supplementary Material 4: Additional file 3. Study of the dose effects of irradiation on the expression of junctions in BBB/BBTB models. Representative images of adherent junctions VE-cadherin and tight-junction-associated proteins Zonula Occludens-1 (ZO-1), Claudin-5 (CLDN-5) 7 days after different doses of irradiation 0, 2, 4, and 6 Gγ on the three models (BBB, BBTB-007 and BBTB-013). Scale bar = 25 µm

Supplementary Material 5: Additional file 4. Investigation of the effects of irradiation on physical properties of the BBTB-013. (A) BBTB-013 ECs permeability to Lucifer Yellow 24 h and 7 days after irradiation (2, 4 and 6 Gγ). (Pe = endothelial permeability coefficient). One-way ANOVA followed by Dunnett’s post-hoc test, ns = non-significant, compared to the non-irradiated control (N = 3; n = 9). (B) Representative images of adherent junctions VE-cadherin and tight-junction-associated proteins zonula occludens-1 (ZO-1), 7 days after 6 Gγ of irradiation. Scale bar = 25 µm. (C) & (D). Protein levels of Claudin-5 and ZO-1 in BBTB-013 ECs, 7 days after irradiation at a dose of 6 Gy, quantified by Western blot and normalized to GAPDH protein level. The line represents the non-irradiated control. Unpaired t-test was used for statistical study, (N = 3)

Supplementary Material 6: Additional file 5. ECs cell count and metabolic activity test. (A) Evolution of endothelial cells numbers 24 h and 7 days after irradiation, as a function of the irradiation dose received 0, 2, 4 and 6 Gγ, for the three models (BBB, BBTB-007 and BBTB-13). The results were expressed as a percentage relative to the non-irradiated control. For the statistical study One-way ANOVA followed by Dunnett’s multiple comparisons test was used. ns = non-significant, *p < 0.5, **p < 0.01, ****p < 0.0001, compared to the non-irradiated control. (N = 3; n = 15). A statistical analysis with outliers was performed in Additional File 7B.) (B) Mitochondrial activity of endothelial cells was assessed by MTT assay, 7 days after irradiation at 6 Gy. Results were expressed as a percentage relative to the non-irradiated control. Positive controls for decreased metabolic activity were performed to validate the MTT assay with using Triton X-100 treatment: an average activity of 3.2% was obtained for the non-irradiated control and 2.5% for the conditions irradiated at a dose of 6 Gγ. Unpaired t-test was used for statistical study, (N = 2; n = 6)

Supplementary Material 7: Additional file 6. Characterization of metabolic properties of BBTB-013 after irradiation: Transcriptional ECs (A). and hBPs (B) genes expression of efflux transporters (P-gp, BCRP, MRP1, MRP2, MRP4 and MRP5), detoxification enzymes (CYP1A1, CYP1B1, CYP2D6, CYP2S1) and transporter MFSD2A, 7 days after irradiation of 2, 4 and 6 Gγ, quantified by RT-qPCR and normalized on the expression of the housekeeping gene GAPDH. For the statistical study One-way ANOVA followed by Dunnett’s multiple comparisons test was used, ns = non-significant *p < 0.05, **p < 0.01, ***p < 0.001, ****p < 0.0001, compared to the non-irradiated control group, (N = 2; n = 6). (C) & (D). Protein levels of efflux transporters P-gp, BCRP, MRP1, MRP4 and MFSD2A (Isoforms (Iso) 1&2), 7 days after irradiation at a dose of 6 Gγ, were quantified by Western blot (upper graph ECs, lower graph hBPs) and normalized to protein level of β-actin. The line represents the level of expression in non-irradiated control. Unpaired t-test was used for statistical study, (N = 3)

Supplementary Material 8: Additional file 7. Comparison of statistical analysis in presence or absence of the outliers. (A) Endothelial permeability to Lucifer Yellow of BBB models 7 days after 2 Gγ of irradiation, in presence or absence of the outliers. One-way ANOVA followed by Dunnett’s post-hoc test, ns = non-significant, compared to the non-irradiated control, (BBB N = 3; n = 9). (B) Endothelial cells numbers expressed in percentage, 7 days after irradiation, as a function of the irradiation dose received 2 and 4 Gγ, for the BBB model, in presence or absence of the outliers. One-way ANOVA followed by Dunnett’s multiple comparisons test was used. **p < 0.01, ****p < 0.0001, compared to the non-irradiated control. (N = 3; n = 15)

## Data Availability

All relevant data are within the paper and its supporting information files.

## Abbreviations

ABCB1: ATP Binding Cassette Subfamily B Member 1 (P-gp)
ABCC: ATP Binding Cassette Subfamily C Member (MRP)
ABCG2: ATP Binding Cassette Subfamily G Member 2 (BCRP)
AGS: Astrocyte Growth Supplement
BSA: Bovin serum albumin
BBB: Blood-brain barrier
BBTB: Blood-brain tumor barrier
BCRP: Breast-Cancer-Resistance Protein
C^14^: ^14^C-Saccharose
cDNA: complementary Deoxyribonucleic Acid
CLDN-5: Claudin-5
CYP: Cytochrome P450
DIPG: Diffuse Intrinsic Pontine Glioma
DHA: DocosaHexaenoic Acid
DSB: Double-strand breaks
DTT: Dithiothreitol
DMEM: Dulbecco’s modified Eagle’s medium
DMG: Diffuse midline glioma
DPM: Decay per minute
ECL: Enhanced chemiluminescent
ECs: Human endothelial cells
EGF: Epidermal growth factor
FCS: Fetal Calf Serum
FGF: Fibroblast growth factor
Gγ: Gray
GAPDH: Glyceraldehyde-3-Phosphate Dehydrogenase
γ-H2AX: Phosphorylated form of H2A histone family member X
HA-bs: Human Astrocytes from brainstem
H3K27: Lysine in position 27 of histone H3
H3K27M: Mutation H3.1K27M or H3.3K27M
hBPs: Human Brain Pericytes
HRP: Horseradish peroxidase
HSA: Human serum albumin
qPCR: Quantitative Polymerase Chain Reaction
LY: Lucifer Yellow
MFSD2A: Major Facilitator Superfamily Domain Containing 2
mRNA: messenger Ribonucleic Acid
MRP: Multidrug-resistance-associated Proteins
PAF: Paraformaldehyde fixative solution
PBS-CMF: Phosphate-Buffered Saline Calcium and Magnesium Free
PDGF: Platelet Derived Growth Factors
PDGFR-β: Platelet Derived Growth Factors Receptor Beta
Pe: Permeability Coefficicent
P-gp: P-glycoprotein
PLL: Poly-L-Lysine
R123: Rhodamine 123
RH: Ringer-HEPES
RPM: Rotor per minute
RT: Room temperature
RT-qPCR: Reverse transcription - quantitative Polymerase Chain Reaction
SBB: Serum Blocking buffer
SV40: Simian Virus 40
TBST: Tris-buffered saline with Tween 20
TNFα: Tumor necrosis factor alpha
ZO-1: Zonula Occludens-1
