# Supplementary figures and images for: Consequences of irradiation on blood-brain tumor barrier model of Diffuse Midline Glioma: characterization of physical and metabolic properties
Source: Fluids Barriers CNS. 2026 Feb 24;23:39. doi: 10.1186/s12987-026-00778-6 (PMC12961863; doi:10.1186/s12987-026-00778-6)

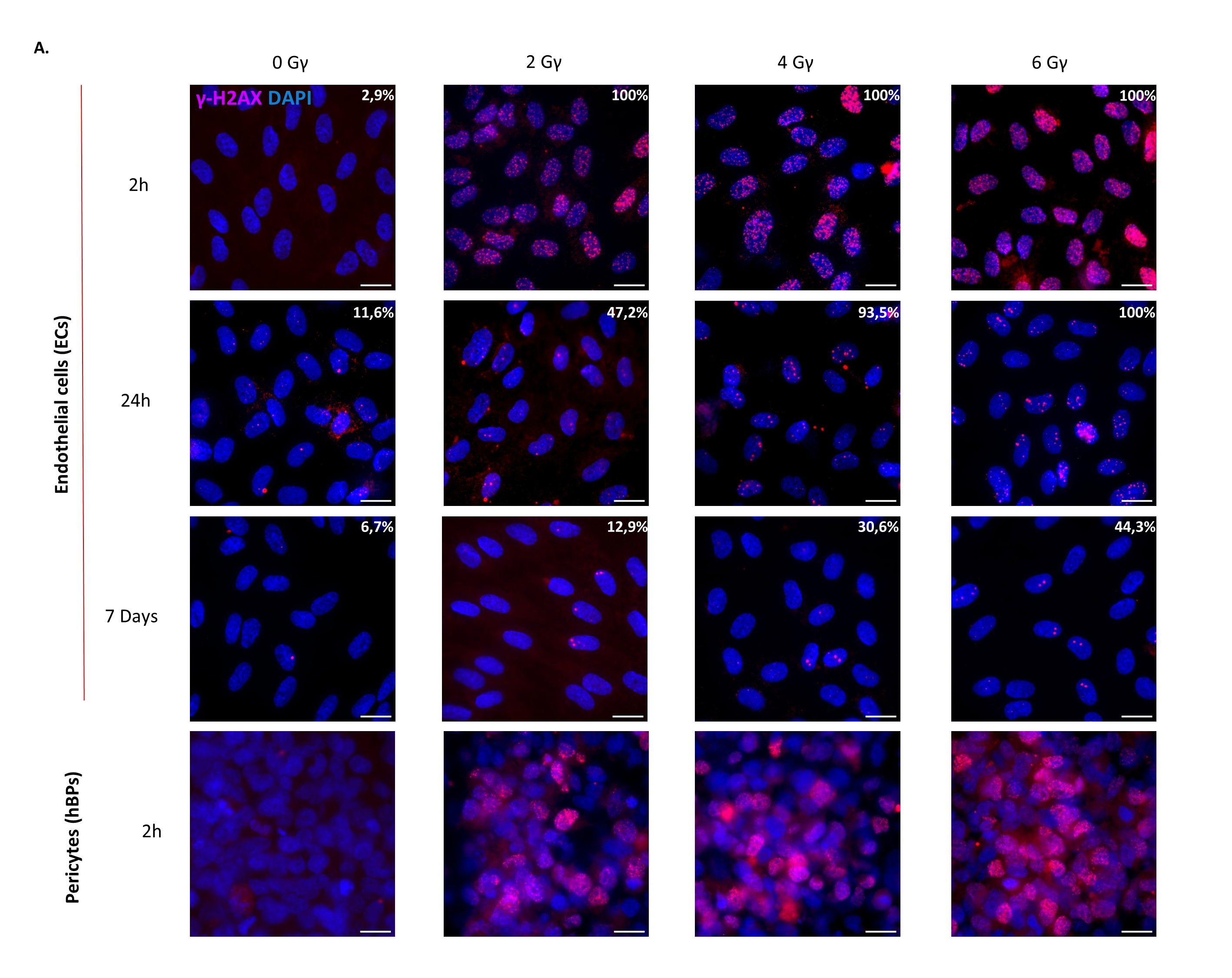

Supplement: Supplementary file 1 — Supplementary Material 1: Additional file 1. Induction of double-strand breaks (DSB) in BBB/ BBTB models. (A) Representative images of immunofluorescent staining of the phosphorylation of histone H2AX (γ-H2AX) to visualize the DSB at 2 h, 24 h and 7 days after irradiation at 0 to 6 Gγ in ECs and on hBPs at 2 h after irradiation dose at 0 to 6 Gγ. On images of ECs, the percentage of γ-H2AX positive cells is indicated. Scale bar = 10 µm. (B) Representative images of γ-H2AX to visualize the DSB at 2 h and 7 days after irradiation at 0 and 6 Gγ in human astrocytes brainstem, HSJD-DIPG-007 and HSJD-DIPG-013 present in the cerebral compartment of the model. Scale bar = 200 µm [file 12987_2026_778_MOESM1_ESM.tif]

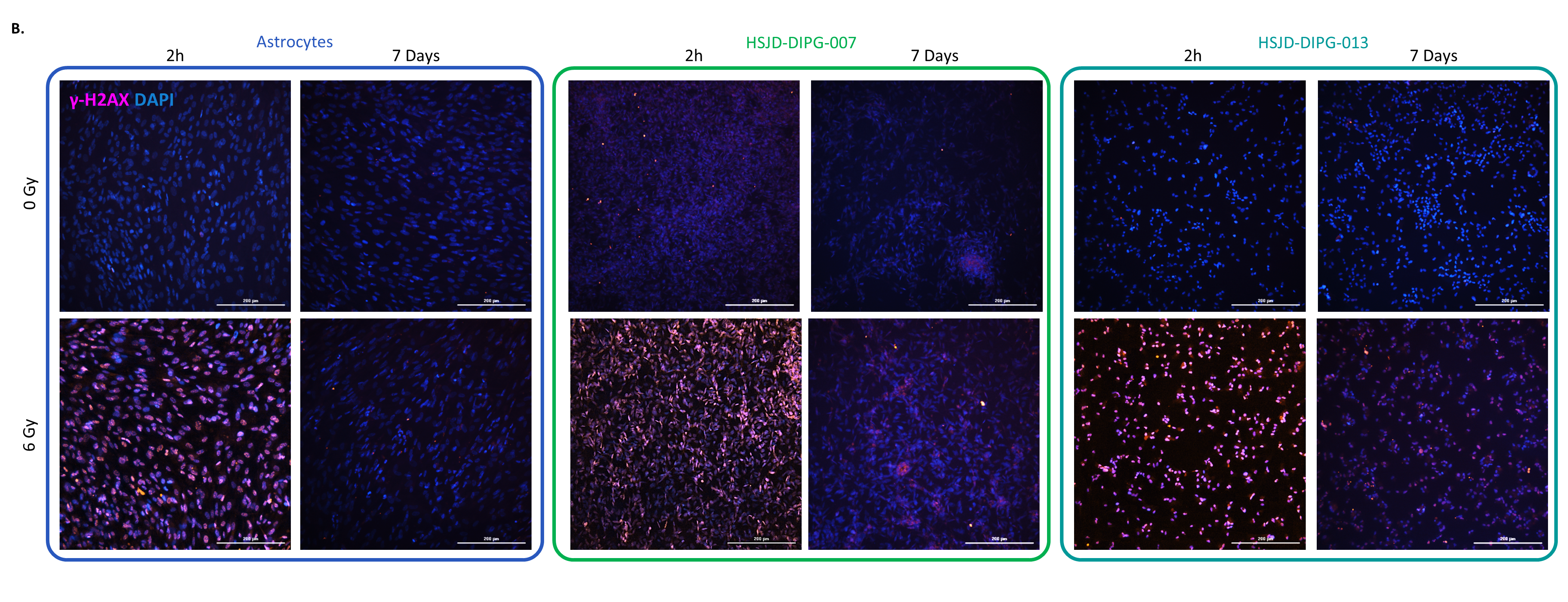

Supplement: Supplementary file 2 — Supplementary Material 2 [file 12987_2026_778_MOESM2_ESM.tif]

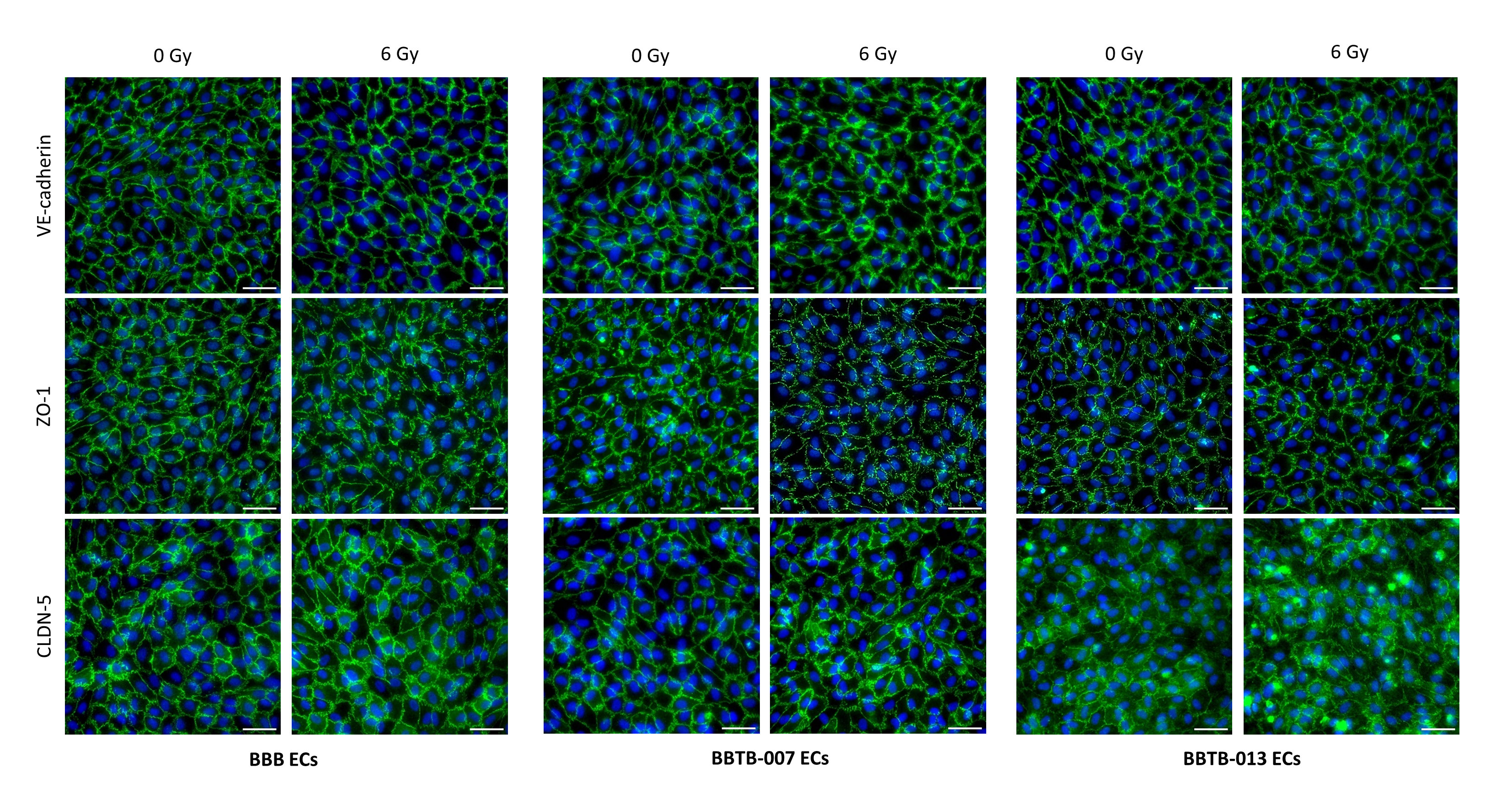

Supplement: Supplementary file 3 — Supplementary Material 3: Additional file 2. Study of the effects of irradiation on the expression of junctions in BBB/BBTB models. Representative images of adherent junctions VE-cadherin and tight-junction-associated proteins Zonula Occludens-1 (ZO-1), Claudin-5 (CLDN-5) 24 h after irradiation dose of 6 Gγ on the three models (BBB, BBTB-007 and BBTB-013). Scale bar = 25 µm [file 12987_2026_778_MOESM3_ESM.tif]

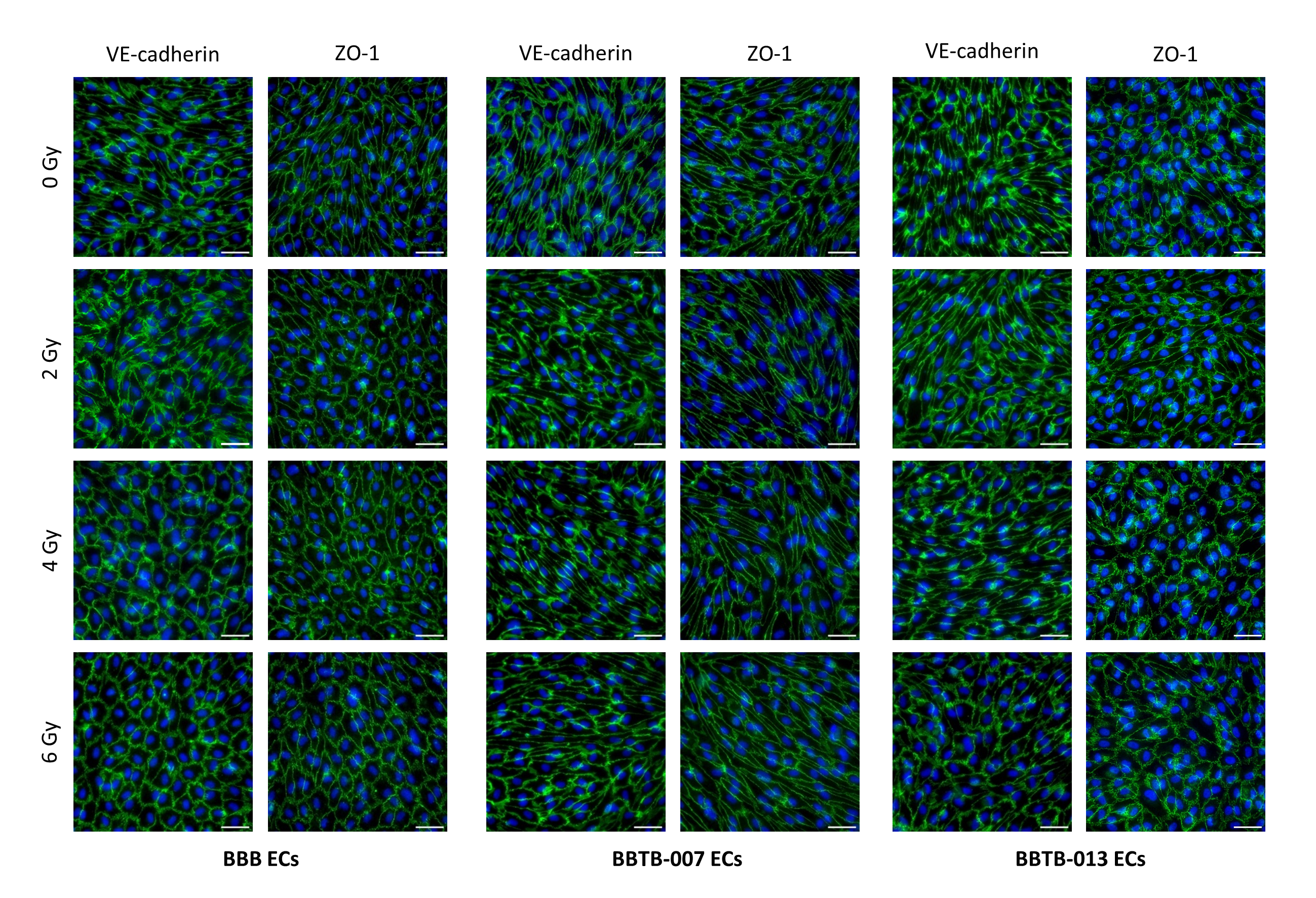

Supplement: Supplementary file 4 — Supplementary Material 4: Additional file 3. Study of the dose effects of irradiation on the expression of junctions in BBB/BBTB models. Representative images of adherent junctions VE-cadherin and tight-junction-associated proteins Zonula Occludens-1 (ZO-1), Claudin-5 (CLDN-5) 7 days after different doses of irradiation 0, 2, 4, and 6 Gγ on the three models (BBB, BBTB-007 and BBTB-013). Scale bar = 25 µm [file 12987_2026_778_MOESM4_ESM.tif]

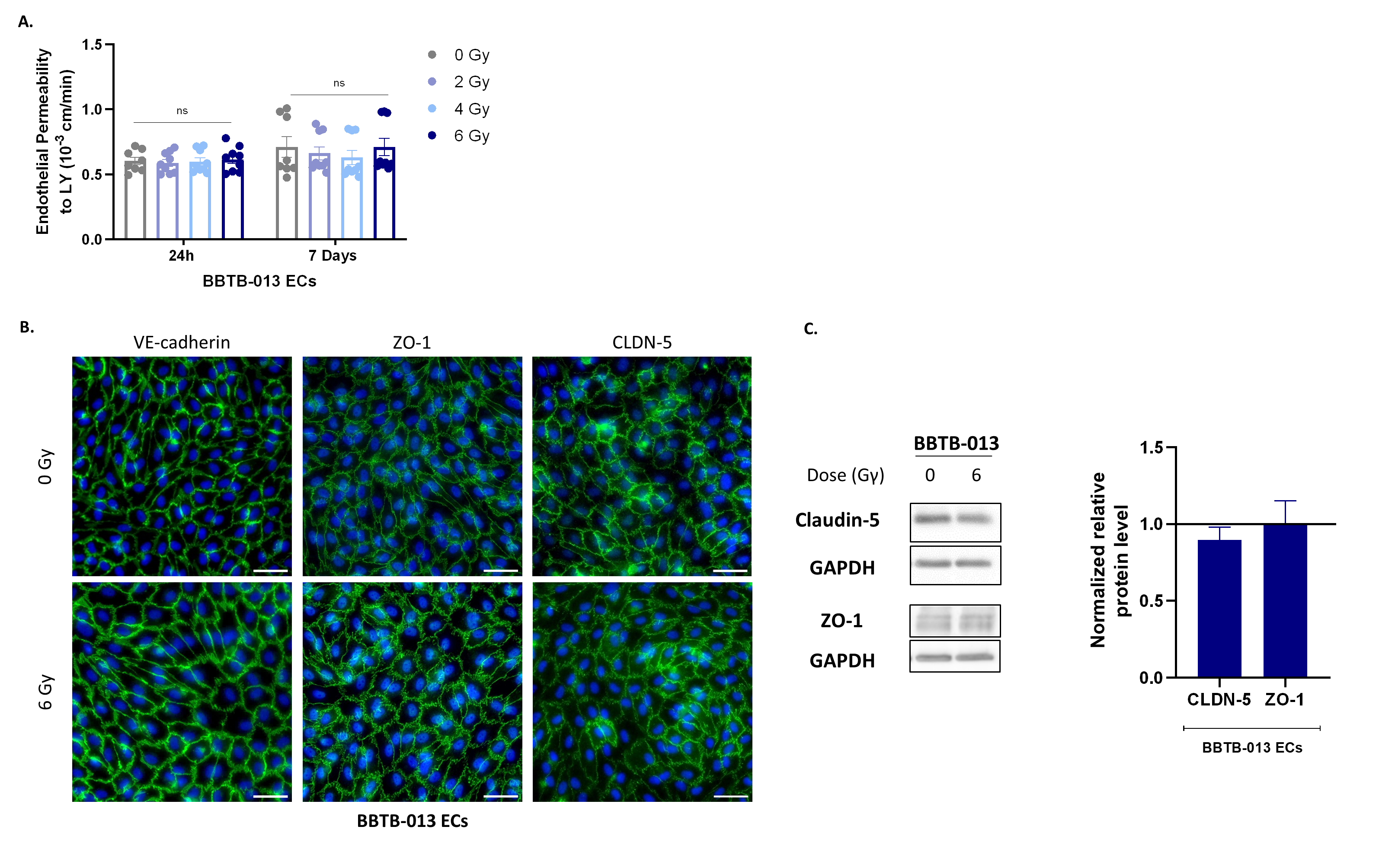

Supplement: Supplementary file 5 — Supplementary Material 5: Additional file 4. Investigation of the effects of irradiation on physical properties of the BBTB-013. (A) BBTB-013 ECs permeability to Lucifer Yellow 24 h and 7 days after irradiation (2, 4 and 6 Gγ). (Pe = endothelial permeability coefficient). One-way ANOVA followed by Dunnett’s post-hoc test, ns = non-significant, compared to the non-irradiated control (N = 3; n = 9). (B) Representative images of adherent junctions VE-cadherin and tight-junction-associated proteins zonula occludens-1 (ZO-1), 7 days after 6 Gγ of irradiation. Scale bar = 25 µm. (C) & (D). Protein levels of Claudin-5 and ZO-1 in BBTB-013 ECs, 7 days after irradiation at a dose of 6 Gy, quantified by Western blot and normalized to GAPDH protein level. The line represents the non-irradiated control. Unpaired t-test was used for statistical study, (N = 3) [file 12987_2026_778_MOESM5_ESM.tif]

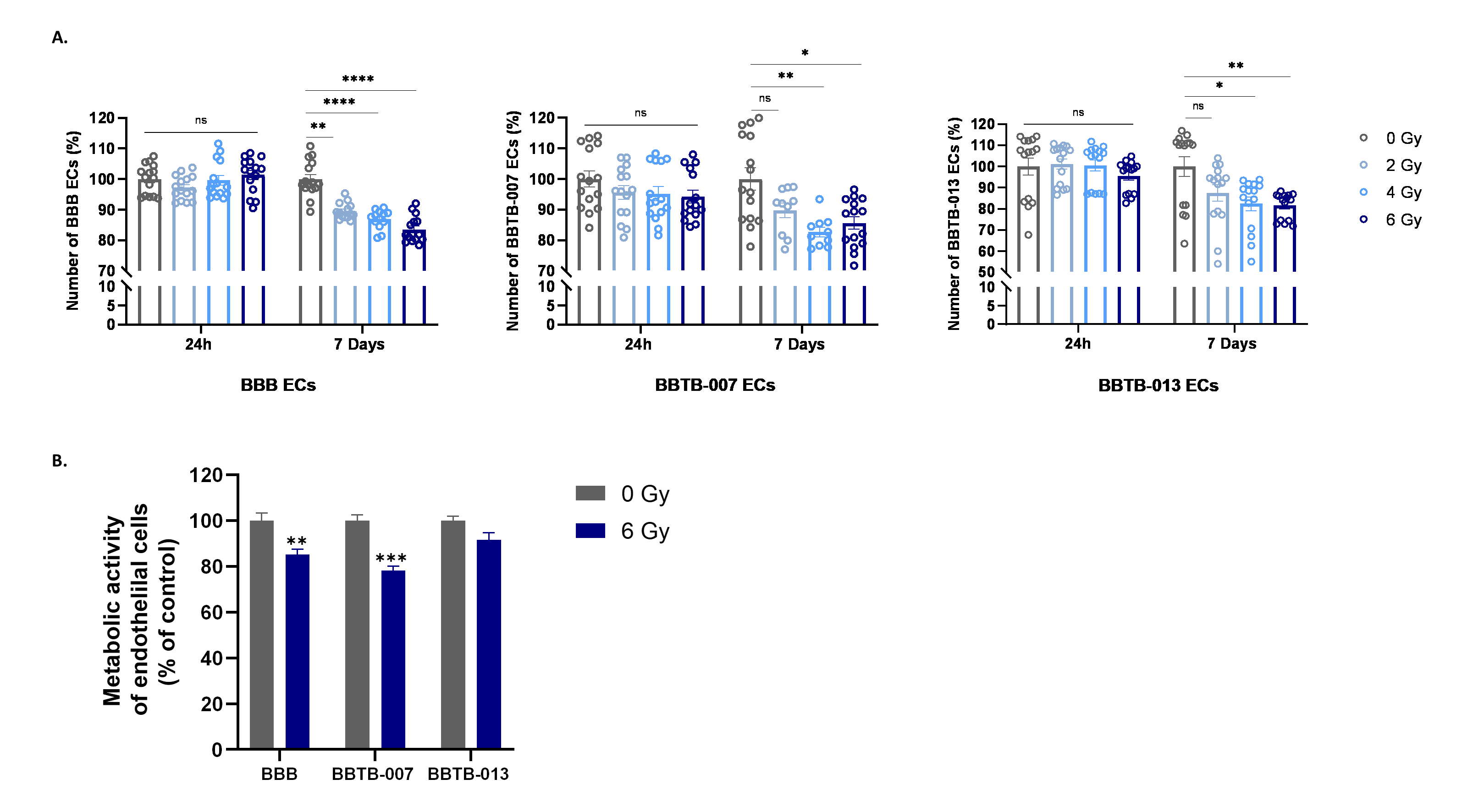

Supplement: Supplementary file 6 — Supplementary Material 6: Additional file 5. ECs cell count and metabolic activity test. (A) Evolution of endothelial cells numbers 24 h and 7 days after irradiation, as a function of the irradiation dose received 0, 2, 4 and 6 Gγ, for the three models (BBB, BBTB-007 and BBTB-13). The results were expressed as a percentage relative to the non-irradiated control. For the statistical study One-way ANOVA followed by Dunnett’s multiple comparisons test was used. ns = non-significant, *p < 0.5, **p < 0.01, ****p < 0.0001, compared to the non-irradiated control. (N = 3; n = 15). A statistical analysis with outliers was performed in Additional File 7B.) (B) Mitochondrial activity of endothelial cells was assessed by MTT assay, 7 days after irradiation at 6 Gy. Results were expressed as a percentage relative to the non-irradiated control. Positive controls for decreased metabolic activity were performed to validate the MTT assay with using Triton X-100 treatment: an average activity of 3.2% was obtained for the non-irradiated control and 2.5% for the conditions irradiated at a dose of 6 Gγ. Unpaired t-test was used for statistical study, (N = 2; n = 6) [file 12987_2026_778_MOESM6_ESM.tif]

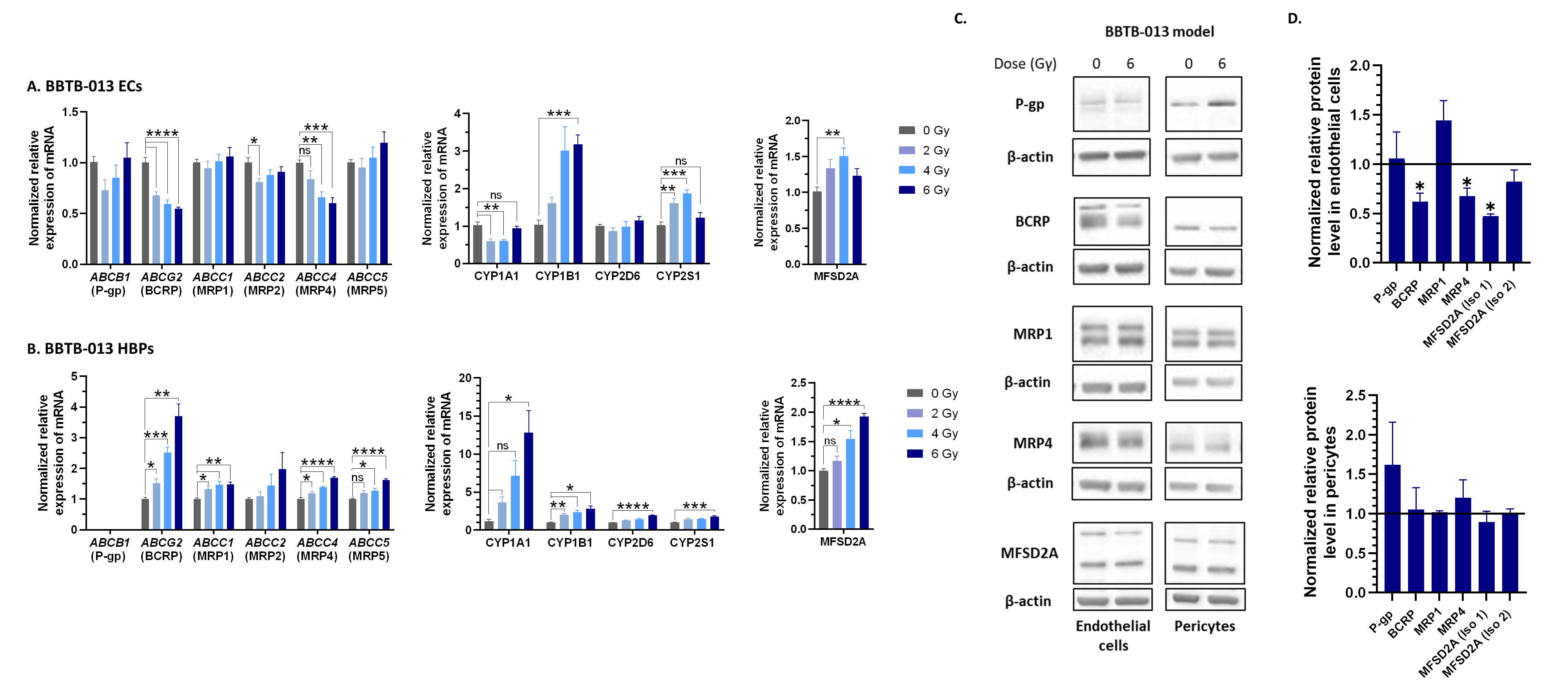

Supplement: Supplementary file 7 — Supplementary Material 7: Additional file 6. Characterization of metabolic properties of BBTB-013 after irradiation: Transcriptional ECs (A). and hBPs (B) genes expression of efflux transporters (P-gp, BCRP, MRP1, MRP2, MRP4 and MRP5), detoxification enzymes (CYP1A1, CYP1B1, CYP2D6, CYP2S1) and transporter MFSD2A, 7 days after irradiation of 2, 4 and 6 Gγ, quantified by RT-qPCR and normalized on the expression of the housekeeping gene GAPDH. For the statistical study One-way ANOVA followed by Dunnett’s multiple comparisons test was used, ns = non-significant *p < 0.05, **p < 0.01, ***p < 0.001, ****p < 0.0001, compared to the non-irradiated control group, (N = 2; n = 6). (C) & (D). Protein levels of efflux transporters P-gp, BCRP, MRP1, MRP4 and MFSD2A (Isoforms (Iso) 1&2), 7 days after irradiation at a dose of 6 Gγ, were quantified by Western blot (upper graph ECs, lower graph hBPs) and normalized to protein level of β-actin. The line represents the level of expression in non-irradiated control. Unpaired t-test was used for statistical study, (N = 3) [file 12987_2026_778_MOESM7_ESM.tif]

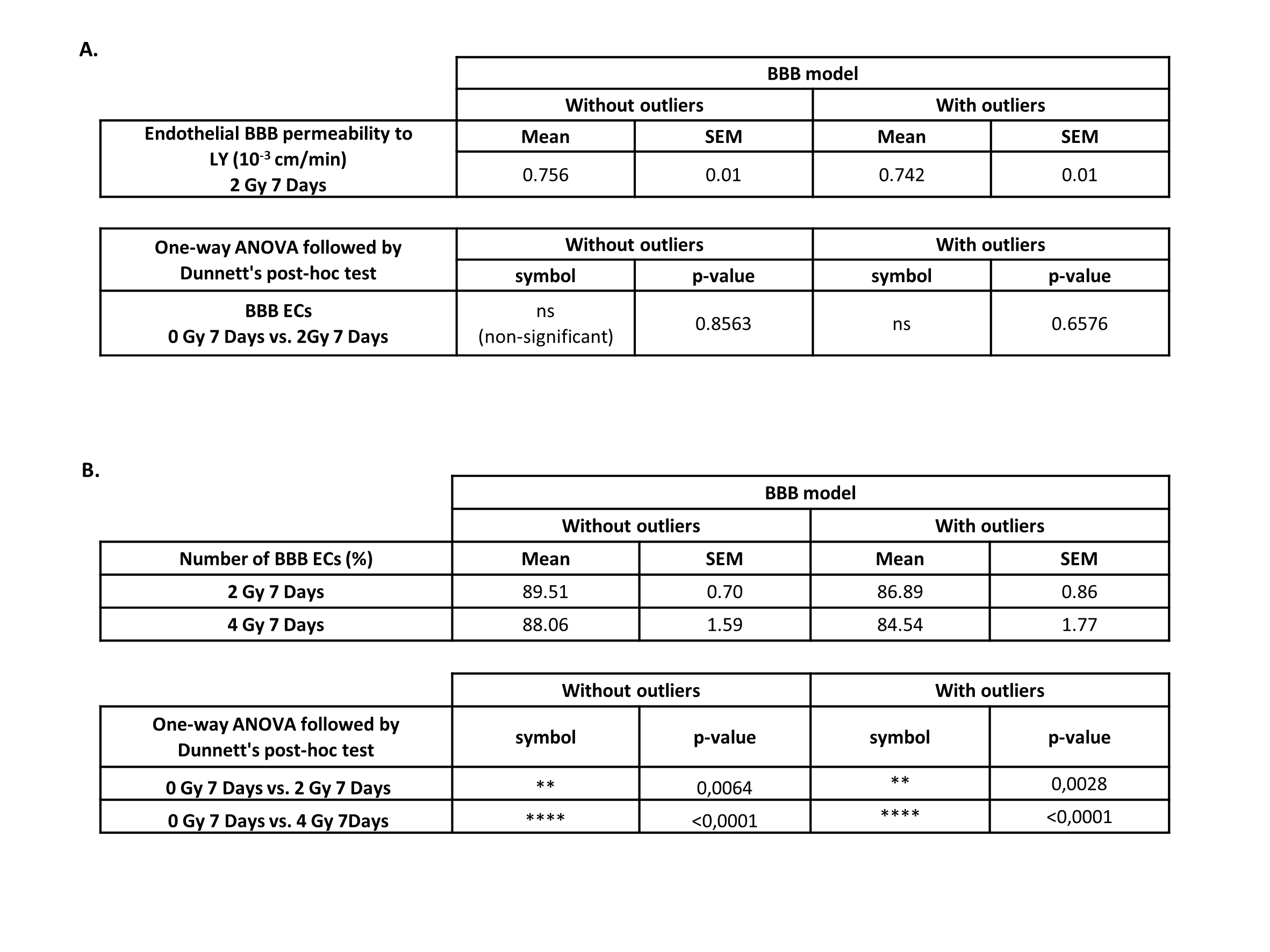

Supplement: Supplementary file 8 — Supplementary Material 8: Additional file 7. Comparison of statistical analysis in presence or absence of the outliers. (A) Endothelial permeability to Lucifer Yellow of BBB models 7 days after 2 Gγ of irradiation, in presence or absence of the outliers. One-way ANOVA followed by Dunnett’s post-hoc test, ns = non-significant, compared to the non-irradiated control, (BBB N = 3; n = 9). (B) Endothelial cells numbers expressed in percentage, 7 days after irradiation, as a function of the irradiation dose received 2 and 4 Gγ, for the BBB model, in presence or absence of the outliers. One-way ANOVA followed by Dunnett’s multiple comparisons test was used. **p < 0.01, ****p < 0.0001, compared to the non-irradiated control. (N = 3; n = 15) [file 12987_2026_778_MOESM8_ESM.tif]
